# Selection in two-sex stage-structured populations: Genetics, demography, and polymorphism

**DOI:** 10.1016/j.tpb.2019.07.012

**Published:** 2019-12

**Authors:** Charlotte de Vries, Hal Caswell

**Affiliations:** University of Amsterdam, Netherlands

**Keywords:** Demography, Genetics, Protected polymorphisms, Two-sex models, Sexual conflict, Eco-evolutionary models

## Abstract

The outcome of natural selection depends on the demographic processes of birth, death, and development. Here, we derive conditions for protected polymorphism in a population characterized by age- or stage-dependent demography with two sexes. We do so using a novel two-sex matrix population model including basic Mendelian genetics (one locus, two alleles, random mating). Selection may operate on survival, growth, or fertility, any or all of which may differ between the sexes. The model can therefore incorporate genes with arbitrary pleiotropic and sex-specific effects. Conditions for protected polymorphism are expressed in terms of the eigenvalues of the linearization of the model at the homozygote boundary equilibria. We show that in the absence of sexual dimorphism, polymorphism requires heterozygote superiority in the genotypic population growth rate. In the presence of sexual dimorphism, however, heterozygote superiority is not required; an inferior heterozygote may invade, reducing the population growth rate and even leading to extinction (so-called evolutionary suicide). Our model makes no assumptions about separation of time scales between ecological and evolutionary processes, and can thus be used to project sex×stage×genotype dynamics of eco-evolutionary processes. Empirical evidence that sexual dimorphism affects extinction risk is growing, yet sex differences are often ignored in evolutionary demography and in eco-evolutionary models. Our analysis highlights the importance of sexual dimorphism and suggests mechanisms by which an allele can be favored by selection, yet drive a population to extinction, as a result of the structure and interdependence of sex- and stage-specific processes.

## 1 Introduction

Among the core results of population genetics are the criteria that determine whether selection leads to fixation of one genetic type, or to coexistence of multiple types in a polymorphism. For viability selection, at a single locus, in discrete generation, random mating, diploid populations, heterozygote advantage in fitness is well known to be a necessary and sufficient condition for a stable polymorphism. However, it is equally well known that the world is populated by species with complex age- or stage-structured life cycles, subject to selection not only on viability but on sex- and stage-specific survival, growth, development, and fertility rates throughout those life cycles.

Original formulations of population genetics included little of the ecological theater that hosts the evolutionary play (Hutchinson, 1965). Fitness in these models was a lumped parameter, tasked with encapsulating all the life cycle processes determining gene transmission from one generation to the next. Temporal variation, multiple niches, density dependence, and age structure were incorporated in due course (e.g., Giesel, 1972, Lewontin and Krakauer, 1973, Waples, 1989, Levene, 1953, MacArthur, 1962, Roughgarden, 1971, Roughgarden, 1979, Charlesworth, 1970, Takada and Nakajima, 1992, Charlesworth, 1994, Takada and Nakajima, 1998). Developments in quantitative genetics (e.g., Lande and Arnold, 1983, Barfield et al., 2011, Coulson and Tuljapurkar, 2008, Coulson et al., 2010) and adaptive dynamics (Metz et al., 1992, Diekmann, 2004) have led to increased ecological realism. Each of these treats genetic processes in its own way. Quantitative genetics considers many genes each with small effects, and adaptive dynamics treats phenotypes, not genotypes.

Much evolutionary theory relies on an implicit or explicit separation of time scales, with ecology assumed to proceed quickly and evolution slowly. It is now recognized that these time scales are not always separated, leading to interest in what has been called ‘eco-evolutionary dynamics’ (Fussmann et al., 2007, Pelletier et al., 2009). Eco-evolutionary studies confront an interdependent set of problems. (1) Time scales: although it is traditional to speak of ecology as acting on short, and evolution on long time scales, some processes operate on the same time scale (e.g., antibiotic and pesticide resistance (Neve et al., 2014), adaptation to urban environments (Schilthuizen, 2018)). This requires a model that explicitly incorporates both processes. (2) Sex structure: gene effects may be sex-specific, because of sexual dimorphism or because they affect processes that are specific to one sex (e.g., lactation by females, courtship behavior by males). This requires a model that distinguishes the two sexes. (3) Stage structure: complex life cycles require more elaborate individual state information than age alone, to accommodate life history characteristics such as maturation, dormancy, dispersal, etc. Genes may have strongly stage-specific effects, and incorporating these requires a model with flexibility in the kinds of structure incorporated. (4) Pleiotropy: genes may affect survival, development, fertility, and stage transitions, all in highly stage-specific ways. Such effects can only be incorporated in a model that explicitly incorporates all those processes. (5) Ecological dynamics: genotypic effects on the vital rates operate together with the ecological and demographic processes influencing those rates, including density effects, resource availability, interspecific interactions, and environmental stochasticity. This requires a model that can incorporate or simplify these effects at will.

In this paper, we derive sufficient conditions for a polymorphism in two-sex, stage-structured populations. In doing so, we develop a model that projects eco-evolutionary dynamics of the sex-stage-genotype structure of a population without requiring any assumptions about separation of ecological and evolutionary time scales. We have purposely chosen to keep the genetics simple (one locus, two alleles, random mating), but to make only minimal assumptions about the ecology and demography of the species. Sex differences in demographic rates can be a consequence of physiological, behavioral, immunological, or morphological differences between the sexes. We use the term sexual dimorphism to refer to sex differences in demographic rates, independent of the driver of those demographic differences.

This paper is organized as follows. First we present the sex× stage×genotype-classified model. We find sufficient conditions for polymorphism by linearizing this model on the homozygous boundaries of the state space, and contrast these conditions with conclusions based on genotype-specific growth rates. We present a hypothetical example that focuses on intra-locus sexual conflict. Finally, we examine special cases that reveal something about the roles of stage structure and sexual dimorphism.

## 2 Constructing two-sex stage-structured genetic models

In this section, we show, step by step, how to derive models that incorporate the life cycle, sexual dimorphism, and genotype dynamics. The construction is intended to be generally applicable. Individuals in the population are jointly classified by sex, stage (1,…,ω), and genotype (1,…,g). Males and females of each genotype may differ in any demographic parameters; these differences among genotypes in stage- and sex-specific demography are raw material for selection.

We make the standard demographic assumption of female demographic dominance, i.e. that enough males are present to fertilize all the females, and the number of offspring produced in a mating is not affected by the stage or genotype (i.e. the i-state) of the male. This assumption could be relaxed by introducing a marriage function (Keyfitz, 1972, Caswell and Weeks, 1986, Schoen, 1988, Shyu and Caswell, 2018), but this is beyond the scope of the current paper.

The vectors describing the population are listed in Table 1. All mathematical objects relating to males are distinguished by a prime (e.g., $n^{\prime }$). To avoid confusion with derivatives, we will write $\frac{d}{dx}$ or $\frac{\partial }{\partial x}$ to indicate (partial) derivatives. The matrix $I_{k}$ is the identity matrix of order k, the vector $1_{k}$ is a k×1 vector of ones, $e_{k}$ is the kth unit vector with a one in the kth entry and zeros elsewhere, $E_{ij}$ is a matrix with 1 in the (i,j)-th entry and zeros elsewhere. The Kronecker product is denoted ⊗ and the vec operator stacks the columns of a matrix to form a vector.

The population vector at t is (1)
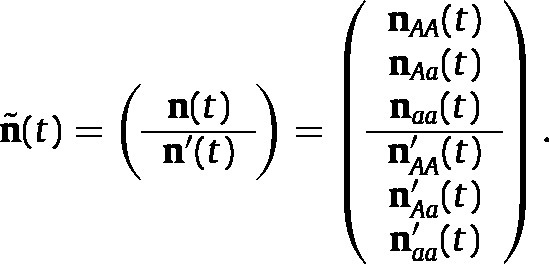
where, e.g., $n_{AA}$ and $n_{AA}^{\prime }$ are the stage distribution vectors of females and males of genotype AA, respectively. The proportional population vector is

(2) $$\overset{\sim }{p}\left(t\right)=\frac{\overset{\sim }{n}\left(t\right)}{\left\|n\left(t\right)\right\|}=\left(\begin{array}{c} p\left(t\right)\\ p^{\prime }\left(t\right) \end{array}\right),$$

where $\left\|\cdot \right\|$ is the 1-norm. The population vector $\overset{\sim }{n}$ is projected from time t to t+1 by an *eco-evolutionary projection matrix* $\overset{\sim }{A}\left[\overset{\sim }{n}\right]$, so that

(3) $$\overset{\sim }{n}\left(t+1\right)=\overset{\sim }{A}\left[\overset{\sim }{n}\left(t\right)\right]\overset{\sim }{n}\left(t\right).$$

The matrix $\overset{\sim }{A}$ is a function of $\overset{\sim }{n}$ because the production of genotypes at t+1 depends on genotype distributions at t.

**Table 1:** Mathematical notation. Dimensions are given where relevant.

Symbol	Definition	Dimension
a	Number of alleles (2 in this case)	
g	Number of genotypes (3 in this case)	
ω	Number of stages	
N	Total population size	
$\overset{\sim }{n}$	Joint sex×genotype×stage vector	2ωg×1
n	Female stage×genotype vector	ωg×1
$n^{\prime }$	Male stage×genotype vector	ωg×1
$\overset{\sim }{p}$	Joint sex×genotype×stage frequency vector	2ωg×1
p	Female stage×genotype frequency vector	ωg×1
$p^{\prime }$	Male stage×genotype frequency vector	ωg×1
$q_{i}$	Allele frequency vector in genotype i	a×1
$q_{b}$	Allele frequency vector in breeding population	a×1

The population projection matrix $\overset{\sim }{A}$ is constructed from a set of matrices that capture the demographic processes for each sex and genotype: See the equations given in Box I.

The $F_{i}$ contain stage-specific fertilities for females of genotype i. The matrices $F_{i}^{\prime }$ determine the contribution by males of genotype i to the gamete pool, and therefore to zygotes in the next generation. A genotype that led to male sterility would result in $F_{i}^{\prime }=0$.

Box I $$\begin{array}{cccc} U_{i} & \text{survival and transitions for females of genotype }i & i=1,\ldots ,g & \omega \times \omega \\ U_{i}^{\prime } & \text{survival and transitions for males of genotype }i & i=1,\ldots ,g & \omega \times \omega \\ F_{i} & \text{fertility matrix for females of genotype }i & i=1,\ldots ,g & \omega \times \omega \\ F_{i}^{\prime } & \text{stage-specific “mating success” matrix for males of genotype }i & i=1,\ldots ,g & \omega \times \omega \end{array}$$

In general these genotype- and sex-specific transition and fertility matrices, $U_{i}$, $U_{i}^{\prime }$, $F_{i}$, and $F_{i}^{\prime }$, could be linear or nonlinear, time-invariant or time varying, deterministic or stochastic, and may include dependence on environmental resources or interactions among species. In this paper we restrict attention to linear, time-invariant demography.

The model formally contains matrices describing the transitions of individuals among genotype classes (Caswell et al., 2018), but since individuals do not change genotype these are identity matrices.

We also define matrices $H_{j}\left(\overset{\sim }{n}\right)$ for j=1,…,ω, of dimension g×g, that assign the offspring of a mother in stage j to the genotypes. The (k,ℓ) entry of $H_{j}$ is the probability that the offspring of a genotype ℓ mother, of stage j, has genotype k. These probabilities depend on the mating frequencies. We assume that mating is random with respect to stage and genotype, and hence that the parent–offspring map is the same for all stages, i.e. $H_{j}\left(\overset{\sim }{n}\right)=H\left(\overset{\sim }{n}\right)$. Assortative mating by stage would lead to differences among the $H_{j}$.

### 2.1 The male contribution to reproduction

The number of offspring is determined by the female genotype, whereas the male genotype determines the contribution of its genes to zygotes in the next time step. We refer to this male contribution as male “mating success” but it may reflect a range of behavioral or physiological characteristics, such as courtship behavior, gamete production, or gamete viability. Males of each stage and genotype combination contribute differentially to a gamete pool. The allele frequencies in the male gamete pool are obtained from the male stage×genotype vector:

(4) $$W^{\prime }F^{\prime }p^{\prime }=\left(\begin{array}{ccc} 1_{\omega }^{T} & \frac{1}{2}1_{\omega }^{T} & 0\\ 0 & \frac{1}{2}1_{\omega }^{T} & 1_{\omega }^{T} \end{array}\right)\left(\begin{array}{ccc} F_{AA}^{\prime } & 0 & 0\\ 0 & F_{Aa}^{\prime } & 0\\ 0 & 0 & F_{aa}^{\prime } \end{array}\right)\left(\begin{array}{c} p_{AA}^{\prime }\\ p_{Aa}^{\prime }\\ p_{aa}^{\prime } \end{array}\right).$$

The matrix $F^{\prime }$ operates on the vector of male genotype frequencies to give the relative contributions of each genotype to the gamete pool. The matrix $W^{\prime }$ converts these relative genotype contributions to allele numbers. Normalizing this vector gives the allele frequencies in the gamete pool,

(5) $$\left(\begin{array}{c} q_{A}^{\prime }\\ q_{a}^{\prime } \end{array}\right)=\frac{W^{\prime }F^{\prime }p^{\prime }}{\left\|W^{\prime }F^{\prime }p^{\prime }\right\|}\;=\;\frac{W^{\prime }F^{\prime }n^{\prime }}{\left\|W^{\prime }F^{\prime }n^{\prime }\right\|}.$$

These frequencies determine the distribution of genotypes in the offspring of a female of any genotype.

### 2.2 Genotype distributions in offspring; the matrix H(n)

From (5) it follows that H(⋅) is a homogeneous of degree zero function of its argument; thus it can be written as a function of either $\overset{\sim }{n}$ or $\overset{\sim }{p}$. The parent–offspring matrix is a function of the allele frequencies in the gamete pool,

(6) $$H\left(\overset{\sim }{n}\right)=\left(\begin{array}{ccc} q_{A}^{\prime } & \frac{1}{2}q_{A}^{\prime } & 0\\ q_{a}^{\prime } & \frac{1}{2} & q_{A}^{\prime }\\ 0 & \frac{1}{2}q_{a}^{\prime } & q_{a}^{\prime } \end{array}\right).$$

The allele frequencies $q_{A}^{\prime }$ and $q_{a}^{\prime }$ are given in terms of either $\overset{\sim }{p}$ or $\overset{\sim }{n}$ by (5). The first column of $H\left(\overset{\sim }{n}\right)$ contains the genotype distribution of the offspring of an AA mother; she produces an AA offspring with probability $q_{A}^{\prime }$ and an Aa offspring with probability $q_{a}^{\prime }$. The second and third columns give the genotype distributions for mothers of genotypes Aa and aa.

## 3 Population projection

The matrix $\overset{\sim }{A}\left[\overset{\sim }{n}\right]$ that projects the eco-evolutionary dynamics is (7)
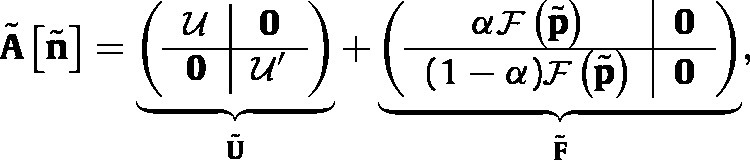
where α is the fraction of newborn individuals that are female and $\overset{\sim }{p}$ is calculated from $\overset{\sim }{n}$ by (2). The blocks correspond to production of females and males by females (αF and (1−α)F in $\overset{\sim }{F}$), and survival of males and females (U and $U^{\prime }$ in $\overset{\sim }{U}$).

To construct $\overset{\sim }{A}$ using the vec-permutation matrix approach (Caswell et al., 2018), create a set of block-diagonal matrices; e.g., 

(8) $$U=\left(\begin{array}{ccc} U_{AA} & 0 & 0\\ 0 & U_{Aa} & 0\\ 0 & 0 & U_{aa} \end{array}\right)$$

and corresponding matrices $U^{\prime }$, F, and $F^{\prime }$. Because individuals do not change their genotype once they are born, the male and female survival matrices are block diagonal; U=U and $U^{\prime }=U^{\prime }$.

Similarly,

(9) $$H\left(\overset{\sim }{p}\right)=I_{\omega }⊗H\left(\overset{\sim }{p}\right)$$

The fertility matrix $F\left(\overset{\sim }{p}\right)$ in (7) is

(10) $$F\left(\overset{\sim }{p}\right)=K^{T}H\left(\overset{\sim }{p}\right)KF.$$

where K is the vec-permutation matrix (Henderson and Searle, 1981). From right to left, the block-diagonal matrix F produces offspring as a function of the genotype of the mother, the vec-permutation matrix K rearranges the vector, the block-diagonal matrix $H\left(\overset{\sim }{n}\right)$ allocates the offspring to their genotypes, and $K^{T}$ returns the vector to its original orientation.

Substituting Eq. (6) into Eq. (10) and simplifying yields (11)
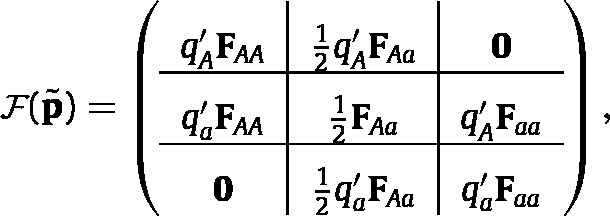
where $q_{A}^{\prime }$ and $q_{a}^{\prime }$ are given by Eq. (5). For a derivation of Eq. (11) from Eq. (10), see de Vries and Caswell (2019b, Appendix A).

Consider the first block column of $F\left(\overset{\sim }{p}\right)$. The first row block produces AA offspring from AA females; this happens when the AA female mates selects allele A from the gamete pool, which happens with probability $q_{A}^{\prime }$. The second row block produces Aa offspring from AA females as a result of selecting allele a from the gamete pool. The other blocks can be interpreted similarly.

Combining all the components yields the eco-evolutionary projection matrix (see Eq. (12) which is given in Box II ),

where $q_{A}^{\prime }$ and $q_{a}^{\prime }$ are given by (5).

Box II

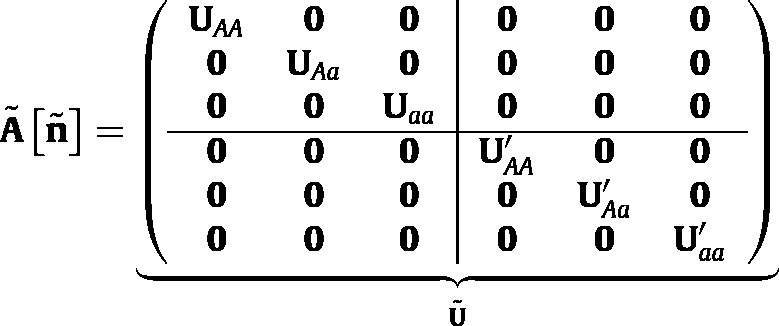

(12)
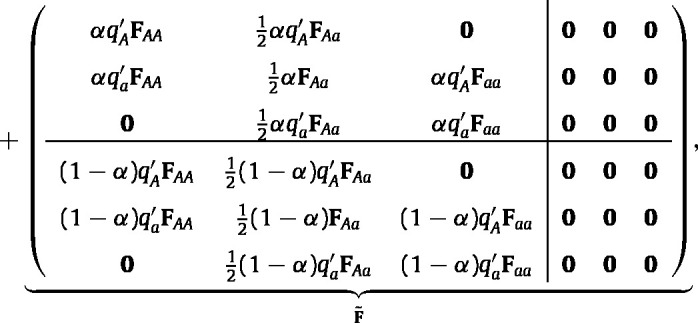

## 4 Conditions for protected polymorphism

The dynamics of $\overset{\sim }{n}$ occur in an ωg-dimensional space defined by combinations of ω stages and g genotypes. The ω-dimensional subspaces defined by the homozygous genotypes AA and aa are referred to as boundaries. These boundaries are invariant under the dynamics specified by $\overset{\sim }{A}\left[\overset{\sim }{n}\right]$. That is, homozygous populations remain homozygous in the absence of mutations.

We approach the problem of allele coexistence by finding sufficient conditions for a protected polymorphism (e.g., Levene, 1953, Prout, 1968, Nagylaki, 1992 Chap. 6). To do so, we calculate the stability of each homozygous population to invasion by the other allele. If both homozygotes are unstable to invasion by the other allele, then the population can never return to a homozygous state, and the genotypes will coexist. We refer to this genotypic coexistence as a protected polymorphism. The conditions for protected polymorphism are determined by linearizing the model in the neighborhood of each boundary equilibrium. This is a lengthy exercise in matrix calculus, the details of which are given in Appendix A.

We emphasize that the dynamics in the interior of the space are generally unknown. Simulations suggest that the frequency vector converges to a unique globally stable equilibrium, but we cannot rule out the possibility of more exotic dynamics. If the ecological component of the model is nonlinear (e.g., density-dependent), then anything is possible.

We will compare the results of the stability analysis with genotype-specific population growth rates, defined as

(13) $$\lambda_{i}=\rho \left(\begin{array}{cc} U_{i}+\alpha F_{i} & 0\\ \left(1-\alpha \right)F_{i} & U_{i}^{\prime } \end{array}\right)\;i=AA,Aa,aa$$

where ρ(⋅) denotes the spectral radius. It is tempting, but ultimately not generally valid, to think of this growth rate as a simple scalar function that, evaluated for each genotype, will reveal its fate. In classical unstructured models under viability selection, λ reduces to the familiar genotypic fitness, and the criterion for a protected polymorphism is heterozygote advantage in fitness. As we will show, heterozygote advantage in λ fails as a criterion for genotype coexistence except in special cases (cf. de Vries and Caswell, 2019b). The reason is that the matrix (13) from which $\lambda_{i}$ is calculated allocates all of the reproduction of genotype i to genotype i, whereas in reality each genotype contributes offspring to other genotypes, depending on the population structure. (The same issue applies to genotype-specific values of the net reproductive rate $R_{0}$ or the reproductive value). Our approach instead is to derive criteria for allele coexistence directly from the matrix $\overset{\sim }{A}\left[\overset{\sim }{n}\right]$ which includes all three genotypes and their interactions.

### 4.1 Boundary equilibria

On a homozygous boundary, (12) reduces to a linear matrix model. Demographic ergodicity guarantees that the homozygous population will converge to a stable stage distribution and grow exponentially; we assume this degree of ergodicity provided the initial population has a nonzero number of females. As in de Vries and Caswell (2019b), we write an equation for the proportional population vector, so that the boundary state is an equilibrium state even if the original population is shrinking or growing,

(14) $$\overset{\sim }{p}\left(t+1\right)=\frac{\overset{\sim }{A}\left[\overset{\sim }{p}\left(t\right)\right]\overset{\sim }{p}\left(t\right)}{\left\|\overset{\sim }{A}\left[\overset{\sim }{p}\left(t\right)\right]\overset{\sim }{p}\left(t\right)\right\|},$$

where $\left\|\cdot \right\|$ is the 1-norm. Equilibrium solutions of (14), denoted by $\overset{ˆ}{p}$, satisfy

(15) $$\overset{ˆ}{p}=\frac{\overset{\sim }{A}\left[\overset{ˆ}{p}\right]\overset{ˆ}{p}}{\left\|\overset{\sim }{A}\left[\overset{ˆ}{p}\right]\overset{ˆ}{p}\right\|}.$$

### 4.2 Linearization at the boundary equilibria

To evaluate the stability of a boundary equilibrium to invasions by the other allele, we linearize (14) in the neighborhood of $\overset{ˆ}{p}$ and determine the spectral radius (magnitude of the largest eigenvalue) of the Jacobian matrix of the linearization. If the spectral radius exceeds one, the boundary equilibrium is unstable. Because $\overset{ˆ}{p}$ is stable to small perturbations within the boundary subspace, a spectral radius larger than one must have an associated eigenvector pointing into the interior, which implies that the invading allele increases when rare.

The Jacobian matrix,

(16) $$M={\frac{\text{d}\overset{\sim }{p}\left(t+1\right)}{\text{d}{\overset{\sim }{p}}^{T}\left(t\right)}|}_{\overset{ˆ}{p}},$$

is obtained by differentiating equation (14) and evaluating the resulting derivative at the boundary equilibrium. The calculations are simplified if i-states in the population vector are arranged by genotype first, then sex, and finally by stage. Then M becomes a block-structured matrix with blocks corresponding to genotypes (17)
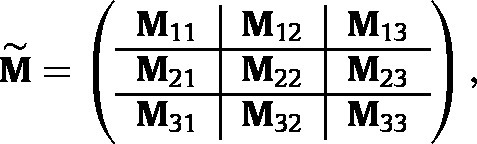
where block $M_{11}$ represents the contribution of perturbations in the AA direction to growth or decline of perturbations in the AA direction, block $M_{12}$ represents the contribution of perturbations in the Aa direction to growth or decline of perturbations in the AA direction, etc. All of the block terms in the Jacobian are given, with their derivation, in Appendix A.

We consider the Jacobian at the AA boundary; the expression at the aa boundary follows by symmetry. At the AA boundary, $\overset{˜}{M}$ is block upper triangular, with $M_{21}=M_{31}=M_{32}=0$; see equation (A-64) in Appendix A.2. Thus the eigenvalues of M are the eigenvalues of the diagonal blocks $M_{11}$, $M_{22}$, and $M_{33}$. Block $M_{11}$ projects perturbations within the AA boundary. Since $\overset{ˆ}{p}$ is stable to such perturbations, the spectral radius of $M_{11}$ must be smaller than one.

Block $M_{33}$ projects perturbations in the aa direction. In the neighborhood of the AA equilibrium, aa homozygotes are negligibly rare, and thus $M_{33}$ normally does not determine the stability of M. An exception occurs when $\lambda_{AA}<\rho \left(U_{aa}\right)<1$. That is, if the AA population is declining sufficiently rapidly, the aa homozygote may increase in frequency simply by declining to extinction more slowly. If the homozygous AA population is stable or increasing, so that $\lambda_{AA}\geq 1$, this cannot happen. Similarly, if $U_{aa}$ is age-classified with a maximum age, $\rho \left(U_{aa}\right)=0$, and the phenomenon cannot happen. We neglect this pathological case in our discussions.

The stability of the AA boundary equilibrium therefore depends on $M_{22}$, which is

(18) $$M_{22}=\frac{1}{\lambda_{AA}}\;\left(\begin{array}{cc} U_{Aa}+\frac{1}{2}\alpha F_{Aa} & \frac{1}{2}\alpha D_{AA}\\ \frac{1}{2}\left(1-\alpha \right)F_{Aa} & U_{Aa}^{\prime }+\frac{1}{2}\left(1-\alpha \right)D_{AA} \end{array}\right)$$

where we define matrices

(19) $$D_{AA}=\frac{\left(F_{AA}{\overset{ˆ}{p}}_{AA}\right)⊗\left(1_{\omega }^{T}F_{Aa}^{\prime }\right)}{1_{\omega }^{T}F_{AA}^{\prime }{\overset{ˆ}{p}}_{AA}^{\prime }}$$

(20) $$D_{aa}=\frac{\left(F_{aa}{\overset{ˆ}{p}}_{aa}\right)⊗\left(1_{\omega }^{T}F_{Aa}^{\prime }\right)}{1_{\omega }^{T}F_{aa}^{\prime }{\overset{ˆ}{p}}_{aa}^{\prime }}$$

and $\lambda_{AA}$ is the AA homozygote population growth rate, given by (13). See equation (A-67) in Appendix A.2 for derivation.

### 4.3 Sufficient conditions for polymorphism

The dominant eigenvalue of the Jacobian matrix at the AA boundary is

(21) $${\overset{\sim }{\zeta }}_{AA}=\frac{1}{\lambda_{AA}}\;\rho \left(\begin{array}{cc} U_{Aa}+\frac{1}{2}\alpha F_{Aa} & \frac{1}{2}\alpha D_{AA}\\ \frac{1}{2}\left(1-\alpha \right)F_{Aa} & U_{Aa}^{\prime }+\frac{1}{2}\left(1-\alpha \right)D_{AA} \end{array}\right).$$

By symmetry, the dominant eigenvalue of the Jacobian matrix at the aa boundary, is

(22) $${\overset{\sim }{\zeta }}_{aa}=\frac{1}{\lambda_{aa}}\;\rho \left(\begin{array}{cc} U_{Aa}+\frac{1}{2}\alpha F_{Aa} & \frac{1}{2}\alpha D_{aa}\\ \frac{1}{2}\left(1-\alpha \right)F_{Aa} & U_{Aa}^{\prime }+\frac{1}{2}\left(1-\alpha \right)D_{aa} \end{array}\right),$$

where $\lambda_{aa}$ is the aa homozygote population growth rate, again given by (13).

Therefore, a protected polymorphism occurs when both boundaries are unstable, i.e. when ${\overset{\sim }{\zeta }}_{AA}>1$ and ${\overset{\sim }{\zeta }}_{aa}>1$, or equivalently when

(23) $$\rho \left(\begin{array}{cc} U_{Aa}+\frac{1}{2}\alpha F_{Aa} & \frac{1}{2}\alpha D_{AA}\\ \frac{1}{2}\left(1-\alpha \right)F_{Aa} & U_{Aa}^{\prime }+\frac{1}{2}\left(1-\alpha \right)D_{AA} \end{array}\right)>\lambda_{AA},$$

(24) $$\rho \left(\begin{array}{cc} U_{Aa}+\frac{1}{2}\alpha F_{Aa} & \frac{1}{2}\alpha D_{aa}\\ \frac{1}{2}\left(1-\alpha \right)F_{Aa} & U_{Aa}^{\prime }+\frac{1}{2}\left(1-\alpha \right)D_{aa} \end{array}\right)>\lambda_{aa}.$$

Eqs. (23), (24) give sufficient conditions for a protected genetic polymorphism for a general two-sex structured population. Eqs. (23), (24) are derived in Appendix A.2, culminating in equations (A-70) and (A-71). The conditions are a function of the demographic rates (through the $U_{i}$, $U_{i}^{\prime }$, $F_{i}$, and $F_{i}^{\prime }$ matrices), the proportion of female newborns, α, and the structure of the homozygote equilibrium, ${\overset{ˆ}{p}}_{AA}$ and ${\overset{ˆ}{p}}_{AA}^{\prime }$ (operating through the matrix $D_{AA}$).

Eqs. (23), (24) are conditions for the instability of the homozygous boundaries, and thus conditions for successful invasion of the boundaries by the heterozygote. One might conjecture that the eigenvalues on the left side of these equations are a measure of the initial heterozygote growth rate during invasion. This is not the case, because a new mutation will appear far from its stable stage distribution, and its initial growth rate will not be given by an eigenvalue.

That said, one might conjecture that the relevance of (23), (24) to the growth rate of a new mutation will depend on the speed with which the population converges to the eigenvector of the linearized dynamics; i.e., on the rate of demographic mixing. If demography operates much faster than genotype dynamics, the heterozygote might achieve temporary demographic stability while still close enough to the boundary that the left-hand side of Eqs. (23), (24) corresponds to the rate of increase of the heterozygote *after* initial transient dynamics/demographic mixing and *before* reaching the coexistence equilibrium. In the language of adaptive dynamics, when evolution is much slower than ecology, the invader will not impact the resident (the demographic structure of the resident is unchanged to first order close to the boundary equilibrium) and we could calculate the growth rate of the invader in the environment set by the resident. This conjecture is an interesting direction for future research.

**Fig. 1:**
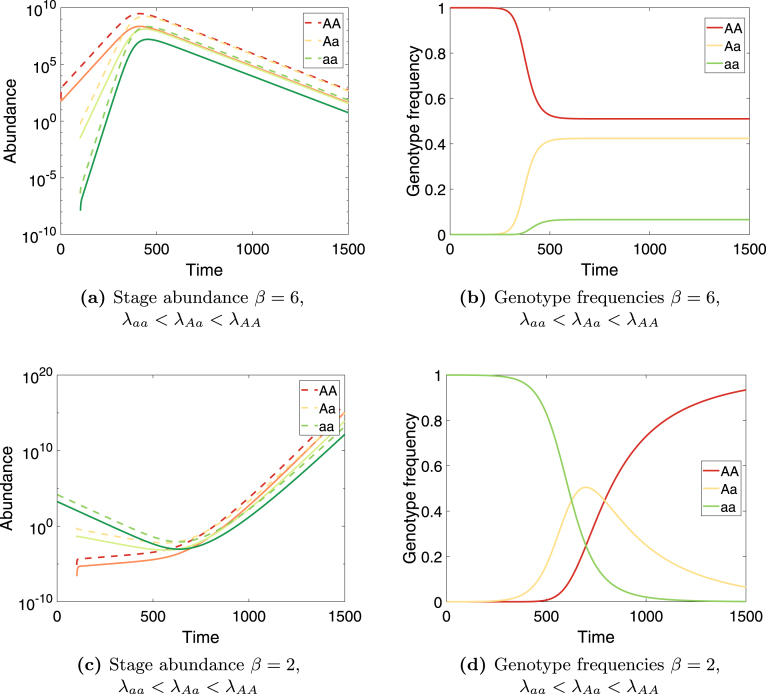
Two examples of population dynamics of a two-sex two-stage Mendelian matrix population model. Dashed lines are juvenile abundances; solid lines are adult abundances. 1a, 1b: Introduction of the a allele leads to a genetic polymorphism and to evolutionary suicide. Male and female population vectors are equal. Parameters used are σ=0.6, s=0.6, γ=0.05, f=8, a=0.6. 1c, 1d: Introduction of the A allele leads to evolutionary rescue and fixation of the AA genotype. Parameters used are σ=0.65, s=0.7, γ=0.05, f=8, a=0.6.

Note that the boundary (in)stability conditions, Eqs. (23), (24), make no assumption about the time scales of ecology and evolution. It is only the conjectured interpretation of the left-hand side of Eqs. (23), (24) as the growth rate of the heterozygote during invasion that requires a separation of ecological and evolutionary time scales. When ecology and evolution operate on similar time scales, the rate of increase of the heterozygote is dominated by transient dynamics until the population reaches its demographic and evolutionary attractor far from the boundary.

## 5 A two-sex projection example: intralocus sexual conflict

Alleles that have differential effects on males and females (sexual dimorphism in allele effects) have important evolutionary consequences. For example, in intralocus sexual conflict, an allele has positive effects on one sex and negative effects on the other. Evidence for such conflict was found by Chippindale et al. (2001) in laboratory-adapted *Drosophila melanogaster*. Genotypes with high male fertilization success tended to have low female fecundity, and vice versa. For a review of studies demonstrating intralocus sexual conflict, see Bonduriansky and Chenoweth (2009).

As an example of our model, we construct and analyze a sex×genotype×stage-classified model for a hypothetical species with intralocus sexual conflict. Our hypothetical species has two life stages: juveniles and adults. Suppose that allele A is beneficial for females but detrimental for males, and that allele a has the reverse effect. As in the *Drosophila* example, suppose that the effects act only during the adult stage through reproductive success or mating success. The allele does not affect survival and transition rates, which we suppose are identical for males and females, such that

(25) $$U_{i}\;=\;U_{i}^{\prime }\;=\;\left(\begin{array}{cc} \sigma \left(1-\gamma \right) & 0\\ \sigma \gamma & s \end{array}\right)$$

for all i. The allele does affect female fertility and male mating success,

(26) $$F_{i}=\left(\begin{array}{cc} 0 & f_{i}\\ 0 & 0 \end{array}\right),$$

(27) $$F_{i}^{\prime }=\left(\begin{array}{cc} 0 & f_{i}^{\prime }\\ 0 & 0 \end{array}\right).$$

and we suppose additive allele effects, so that $f_{i}$ and $f_{i}^{\prime }$ are

**Figure:**
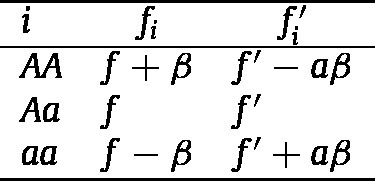

Finally, we assume the sex ratio at birth is even (α=0.5).

Iterating equation (3) with the above demographic matrices provides numerical solutions for selection operating on any sex- and/or stage-specific demographic parameters. Fig. 1 shows two examples of such genotype dynamics during an invasion (Matlab code is given in the Online Supplementary Materials). In Figs. 1a, 1b the a allele is introduced into an AA population after a hundred days. The frequency of the a allele, which reduces female fertility, increases until a genetic polymorphism is reached. Since aa individuals have much lower fertility than the resident AA individuals, the population goes from positive to negative growth rate as a increases in frequency (evolutionary suicide). In Figs. 1c, 1d allele A is introduced into an aa population. The frequency of the A allele increases and fixation of the AA genotype results. As the frequency of the A allele increases, the population moves from negative to positive growth rate (evolutionary rescue).

**Fig. 2:**
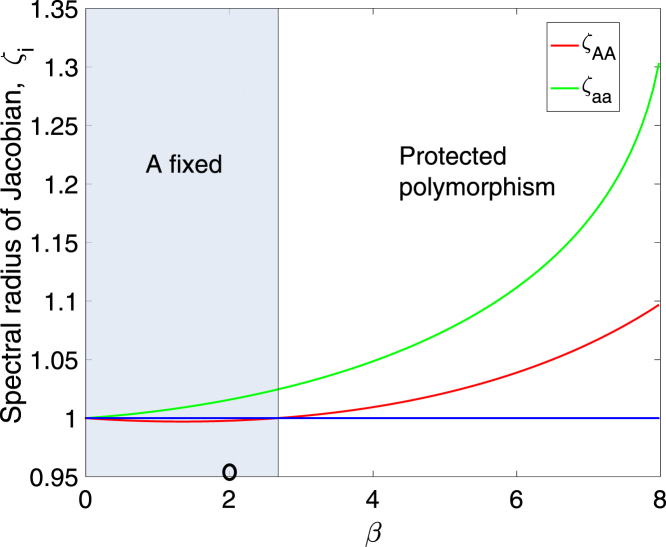
The effect of sexual antagonism on polymorphism stability in the model of Section 5. For β⪅2.7 (the gray area), the AA boundary is stable and the A allele is fixed. For larger values of β, both boundaries are unstable (spectral radius of the Jacobian is larger than one) and a protected polymorphism exists. The open circle indicates parameter values used in Figs. 1c and 1d. Parameters are σ=0.65, s=0.7, γ=0.05, f=8, a=0.6. The population is exponentially growing for all values of β shown.

Armed with the coexistence conditions in Eqs. (23), (24), we calculate how the boundary stabilities (the largest eigenvalue of the Jacobian matrix evaluated at both boundaries) change as a function of the parameter β, which measures the degree of sexual antagonism (large β means a large positive effect on one sex and a large negative effect on the other sex). Fig. 2 shows results when effects on females are larger than those on males (a=0.6). Parameters used are the same as the parameters used in Figs. 1c and 1d, except for β which is plotted on the x-axis. The population is exponentially growing for all values of β. For small values of β allele A fixates from any initial condition (except for starting exactly on the aa boundary). A protected polymorphism exists for values of β≳2.7, as shown in Fig. 2. Larger values of β increase the fecundity of AA females, which increases the growth rate of the AA population on the boundary, but it also benefits an invading a allele because matings between Aa males and AA females create new heterozygotes.

When effects on males and females are equal (a=1), we found a protected polymorphism for any value β. Although this model is simple, the result suggests that sexual antagonism in a sexually dimorphic species can contribute to the maintenance of genetic variation. Chippindale et al. (2001) found that there are indeed extensive genome-wide polymorphisms for sexually antagonistic alleles in *Drosophila*.

## 6 Sexual dimorphism and stage structure

**Fig. 3:**
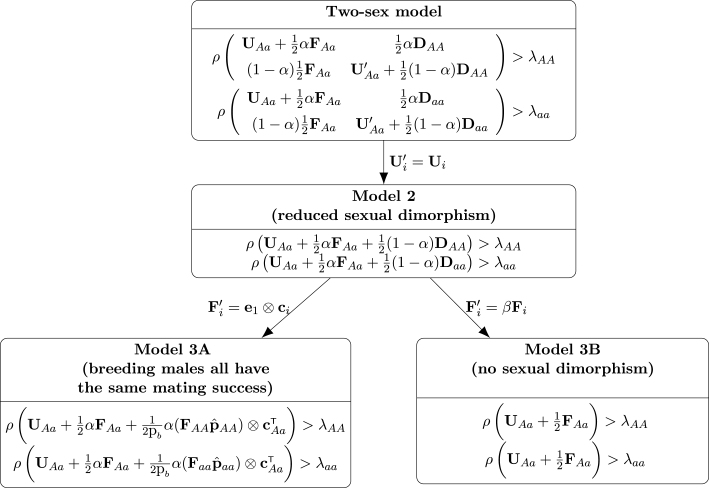
Coexistence conditions for the two-sex model and several modifications that reduce sexual dimorphism. The matrices $D_{AA}$ and $D_{aa}$ are given by (19), (20) and the genotype-specific growth rates are given by (13).

Distinguishing males and females makes it possible to analyze genes with differential effects on the sexes. In Fig. 3 the criteria for polymorphism are shown for increasingly simplifying assumptions about the two sexes. When sexual dimorphism in survival and transition probabilities is eliminated ($U_{i}=U_{i}^{\prime }$ for all i; Model 2 in Fig. 3), the genotype-specific population growth rates (13) simplify to

(28) $$\lambda_{i}=\rho \left(U_{i}+\alpha F_{i}\right)$$

The male and female population vectors are equal at equilibrium under this assumption. If in addition α=1∕2, then starting from any initial vector with nonzero numbers of males and females, the male and female population vectors will be identical once the last individual from the initial cohort has died.^1^

When sexual dimorphism is reduced further, by making male mating success proportional to female fertility (Model 3B in Fig. 3), the conditions (23), (24) for a protected polymorphism reduce to

(29) $$\rho \left(U_{Aa}+\frac{1}{2}F_{Aa}\right)>\rho \left(U_{AA}+\alpha F_{AA}\right)$$

(30) $$\rho \left(U_{Aa}+\frac{1}{2}F_{Aa}\right)>\rho \left(U_{aa}+\alpha F_{aa}\right).$$

See Appendix B.2 for details. If the female proportion $\alpha =\frac{1}{2}$, then (28) implies that the conditions (29), (30) reduce to heterozygote superiority in genotype-specific growth rate, $\lambda_{i}$.

Because α occurs only on the right-hand side of these equations, reducing α reduces the stability of the boundaries and extends the parameter range for which a protected polymorphism is obtained. That is, when females are rare (α is small) a heterozygote may invade even when it is worse at reproducing and surviving than both homozygotes.

In one-sex population genetic models, the gamete pool is constructed from the female population vector. In the case of structured one-sex population genetics models, it is (implicitly) assumed that each (st)age contributes to the gamete pool proportional to its relative abundance in the population (Coulson et al., 2011, de Vries and Caswell, 2019b). The construction of such a one-sex model requires two assumptions: (1) the male and female population vectors are proportional, and (2) male mating success is independent of (st)age and genotype. To describe the mating population, define a vector $c_{j}$ whose entries are 1 if that stage of genotype j reproduces, and 0 otherwise. The male mating success matrix then becomes

(31) $$F_{j}=e_{1}⊗c_{j}^{T}.$$

We refer to this as Model 3A and the conditions for a genetic polymorphism are shown in Fig. 3.

The results in Fig. 3 are valid for any age or stage structure. If structure is eliminated, the matrices $U_{i}$, $F_{i}$, etc. reduce to scalars $u_{i}$, $f_{i}$, etc., but the pattern of the conditions for polymorphism remain; see Figure A1 in Appendix B.

## 7 Discussion

Our main results (Eqs. (23), (24) in Section 4.3) give sufficient conditions for the instability of the boundary equilibria, and thus for a protected polymorphism for sexually dimorphic populations in which selection operates on any demographic rates. The conditions depend on the $M_{22}$ submatrix of the Jacobian. These conditions are also necessary for instability of the boundaries if $\lambda_{AA}>1$ and $\lambda_{aa}>1$.

Sexual dimorphism in survival, fertility, growth, maturation, and other vital rates has been known and studied since Darwin (1888). Two-sex demographic models for population dynamics have been extensively studied (e.g., Keyfitz, 1972, Caswell and Weeks, 1986, Pollak, 1990, Vamosi and Otto, 2002, Iannelli et al., 2005, Jenouvrier et al., 2010, Plard et al., 2018), as have unstructured models for the evolutionary consequences of sexual dimorphism (Owen, 1953, Bodmer, 1965, Feldman et al., 1983, Slatkin, 1984, Bolnick and Doebeli, 2003). Our framework incorporates the ecology and evolution of both sexes for arbitrary life cycle structures.

We have focused on the conditions required for protected polymorphism. These conditions involve the eigenvalues of the linearization near the homozygote boundary equilibria. In general these conditions do not correspond to the genotype-specific population growth rates $\lambda_{i}$. Sexual dimorphism can therefore make it possible for a heterozygote with a lower population growth rate to invade both homozygote boundaries. This can reduce population growth, and can even lead to extinction (so-called evolutionary suicide; see Fig. 1a). Interestingly, using fossil records of ostracods, Martins et al. (2018) recently found that species with greater sexual dimorphism exhibit an increased risk of extinction. Similarly, Hasegawa and Arai (2018) estimated perceived extinction risk in 72 species of swallow and found a higher extinction risk for swallows with sexually dimorphic plumage than for species with sexually monomorphic plumage.

Our analysis suggests many mechanisms that could lead to evolutionary suicide. It is well known that intralocus sexual conflict can do so (Kokko and Brooks, 2003). We also find that an allele with a higher survival for both males and females but a lower (female) fertility can successfully invade a resident population by taking advantage of the residents’ higher fertility during invasion. Once a polymorphism containing this allele is reached, however, its low fertility can push the population from a positive to a negative growth rate (see de Vries and Caswell (2019b)). Similarly, an allele with a faster maturation rate for both sexes at the cost of lower fertility can lead to evolutionary suicide if survival in the adult stage is much higher than in the juvenile stage (unpublished results). Finally, genes that increase male mating success at the expense of offspring survival can also lead to evolutionary suicide (the “Trojan gene hypothesis” of Muir and Howard, 1999).

Kokko and Jennions (2014) define sexual conflict as the existence of a mechanism that allows individuals of sex A to alter what individuals of sex B do at a cost to sex B, and with a selective benefit to sex A (the authors refer to such a mechanism as a ‘tool’). The above mentioned mechanisms for deterministic suicide all fall within this definition of sexual conflict, since they involve a form of intralocus sexual conflict where either both males and females get a benefit but only females pay a cost, or only males get a benefit and females pay the cost (the ‘tool’ in this case is the gene that affects the demographic rates of both sexes). When there is no sexual dimorphism in demography, the interests of males and females are aligned. In this case only genes that improve survival, increase maturation, or increase fertility, i.e., genes that increase population growth rate, can invade.

We have explored a few simplifications of a two-sex stage-structured model in Section 6. Under some conditions, the criteria for polymorphism reduce to heterozygote advantage in the genotype-specific growth rate. This result provides an easily calculated demographic quantity that works the same way as fitness in classical population genetic models. However, the hope for such a general scalar measure of fitness seems a chimera. The net reproductive rate and the reproductive value have the same problems as the genotype growth rate: genotypes do not produce copies of themselves only.

### Some historical context

Effects of sexual dimorphism are already apparent in unstructured models of fertility selection (e.g., Penrose, 1947, Owen, 1953, Bodmer, 1965, Pollak, 1978, Hadeler and Liberman, 1975, Feldman et al., 1983). For example, both Pollak (1978) and Clark and Feldman (1986) found that mean fitness in the population does not always increase when genotypes differ in fertility as well as survival rates. We have found that this result is still valid in the context of a structured population genetic model.

A few papers have previously addressed evolution in two-sex structured populations. Shyu and Caswell (2016b) investigated sex ratio evolution with multiple maternal conditions by combining nonlinear matrix models with multidimensional adaptive dynamics (see also (Shyu and Caswell, 2016a)). Childs et al. (2016) combine a two-sex demographic model with quantitative genetics and extend the age-structured Price equation to breeding values and two sexes. Harts et al. (2014) used an individual based, spatially structured model with dispersing juveniles and female demographic dominance to model local adaptation subjected to intralocus sexual conflict and environmentally driven sex ratio biases.

### Extensions

Our step by step construction of the projection matrix $\overset{\sim }{A}\left[\overset{\sim }{n}\right]$ makes it possible to extend the model in a variety of ways. The demographic components ($U_{i}$, $F_{i}$ and their male counterparts) can be made density-dependent; conditions for polymorphism can still be obtained by linearizing at boundary equilibria (de Vries and Caswell, 2019a). Or the demography could be made time-varying, or environment-dependent. The eco-evolutionary projections would remain unchanged, but the conditions for polymorphism would be more complicated.

In species with biparental care, the characteristics of both parents are known to be important for offspring survival (Sheldon, 2000, Badyaev and Hill, 2002, Rankin and Kokko, 2007). Developing a version of the model that explicitly includes pair formation by incorporating a marriage function (Keyfitz, 1972, Caswell, 2001, Shyu and Caswell, 2018) would make it possible to analyze the genetics of traits related to parental care.

Distortions of the operational sex ratio can lead to limitation of female reproduction by male availability (a “marriage squeeze”) (Schoen, 1983, Goldman et al., 1984). Species from a wide range of taxa have adult sex ratios substantially different from one (Altman et al., 1962, Willson, 1983, Székely et al., 2014), which is likely to result in marriage squeezes. Modeling species with marriage squeezes would require incorporating a marriage function into the model presented here. The flexibility of the model framework introduced here makes these and other extensions possible.

Assortative mating by stage can be included by modifying the mother–offspring map, $H\left(\overset{\sim }{n}\right)$. That is, if females of different stages differ in their mate preference, then the mother–offspring matrices are different for each stage, such that $H_{j}\left(\overset{\sim }{n}\right)\neq H\left(\overset{\sim }{n}\right)$ for j=1,…,ω. If females of different genotypes have different mating preferences, then male mating success depends on the females genotype. This can be implemented by creating a mating success matrix, $F_{i}^{\prime }$ for each female genotype (i=AA,Aa,aa). As a consequence, females of different genotypes interact with a different male gene pool, i.e. the frequencies $q_{A}^{\prime }$ and $q_{a}^{\prime }$ are different in each column of $H\left(\overset{\sim }{n}\right)$.

## Appendix A Supplementary data

MMC S1

## Footnotes

1 For an age-structured population where everybody dies after ω time steps, the male and female population vectors are equal after ω time steps. For a model with an open ended last age class or final life stage, the two vectors asymptotically approach each other.
