# Supplementary material for: Selection in two-sex stage-structured populations: Genetics, demography, and polymorphism
Source: Theor Popul Biol. 2019 Dec;130:160–9. doi: 10.1016/j.tpb.2019.07.012 (PMC6892267; doi:10.1016/j.tpb.2019.07.012)
Supplement: MMC S1 [file mmc1.pdf]

A Appendix: Coexistence conditions

The conditions for a protected polymorphism in the two-sex model, equations (23) and (24), will be derived in this Appendix. The derivation is simplest when the population vector is ordered by genotype first, and then by sex and stage, in contrast to the ordering used in the main text (sex, then genotype, then stage). We will first construct the population projection matrix and then use it to derive coexistence conditions, following closely the logic introduced by de Vries and Caswell (2019).

A.1 Population projection matrix

The population vector is

$$\tilde{\mathbf{n}} = \left(\begin{array}{c} \mathbf{n}_{AA} \\ \mathbf{n}'_{AA} \\ \hline \mathbf{n}_{Aa} \\ \mathbf{n}'_{Aa} \\ \hline \mathbf{n}_{aa} \\ \mathbf{n}'_{aa} \end{array} \right). \tag{A-1}$$

The population projection matrix $\tilde{\mathbf{A}}$ consists of 3×3 blocks, which act on the genotype-specific population vectors:

$$\tilde{\mathbf{A}} = \tilde{\mathbf{U}} + \tilde{\mathbf{F}} \tag{A-2}$$

$$= \left(\begin{array}{cc|cc|cc} \mathbf{U}_{AA} & \mathbf{0} & \mathbf{0} & \mathbf{0} & \mathbf{0} & \mathbf{0} \\ \mathbf{0} & \mathbf{U}'_{AA} & \mathbf{0} & \mathbf{0} & \mathbf{0} & \mathbf{0} \\ \hline \mathbf{0} & \mathbf{0} & \mathbf{U}_{Aa} & \mathbf{0} & \mathbf{0} & \mathbf{0} \\ \mathbf{0} & \mathbf{0} & \mathbf{0} & \mathbf{U}'_{Aa} & \mathbf{0} & \mathbf{0} \\ \hline \mathbf{0} & \mathbf{0} & \mathbf{0} & \mathbf{0} & \mathbf{U}_{aa} & \mathbf{0} \\ \mathbf{0} & \mathbf{0} & \mathbf{0} & \mathbf{0} & \mathbf{0} & \mathbf{U}'_{aa} \end{array} \right)$$

$$+ \left(\begin{array}{cc|cc|cc} q'_A \alpha \mathbf{F}_{AA} & \mathbf{0} & \frac{1}{2} q'_A \alpha \mathbf{F}_{Aa} & \mathbf{0} & \mathbf{0} & \mathbf{0} \\ (1-\alpha) q'_A \mathbf{F}_{AA} & \mathbf{0} & \frac{1}{2}(1-\alpha) q'_A \mathbf{F}_{Aa} & \mathbf{0} & \mathbf{0} & \mathbf{0} \\ \hline q'_a \alpha \mathbf{F}_{AA} & \mathbf{0} & \frac{1}{2} \alpha \mathbf{F}_{Aa} & \mathbf{0} & q'_A \alpha \mathbf{F}_{aa} & \mathbf{0} \\ (1-\alpha) q'_a \mathbf{F}_{AA} & \mathbf{0} & \frac{1}{2}(1-\alpha) \mathbf{F}_{Aa} & \mathbf{0} & (1-\alpha) q'_A \mathbf{F}_{aa} & \mathbf{0} \\ \hline \mathbf{0} & \mathbf{0} & \frac{1}{2} q'_a \alpha \mathbf{F}_{Aa} & \mathbf{0} & q'_a \alpha \mathbf{F}_{aa} & \mathbf{0} \\ \mathbf{0} & \mathbf{0} & \frac{1}{2}(1-\alpha) q'_a \mathbf{F}_{Aa} & \mathbf{0} & (1-\alpha) q'_a \mathbf{F}_{aa} & \mathbf{0} \end{array} \right). \tag{A-3}$$

with symbols defined in the main text. Male and female offspring are produced in a fixed ratio of $\alpha : (1-\alpha)$. The survival matrices appear on the diagonal because individuals do not change their genotype once they are born. The fertility matrix incorporates the Mendelian inheritance and is an extension of the fertility matrix derived in de Vries and Caswell (2019).

The first block column of $\tilde{\mathbf{A}}$ describes the production of offspring by an AA female with stage-specific fertility rates $\mathbf{F}_{AA}$. The probability of picking an A allele out of the gamete pool, and hence the probability of this AA female producing an AA offspring, is q'_A, as derived in the main text. Conversely, the probability of picking an a allele and producing Aa offspring is q'_a. Similarly, the middle column of block matrices are offspring produced by Aa females, which can produce offspring of all 3 genotypes.

A.2 Coexistence conditions

The two-sex Mendelian matrix model defined by equation (A-3) reduces to a linear matrix model on the boundary (since $q'_A = 1$ and $q'_a = 0$). Provided the initial population contains a nonzero number of females, the population will grow or shrink exponentially after converging

to a stable population structure (see Caswell (2001), section 4.5.2.1). Taking advantage of the homogeneity of $\tilde{\mathbf{F}}$, we rewrite the model in terms of the normalized population vector (the frequency vector):

$$\tilde{\mathbf{p}}(t+1) = \frac{\tilde{\mathbf{A}}[\tilde{\mathbf{p}}(t)]\tilde{\mathbf{p}}(t)}{\left\|\tilde{\mathbf{A}}[\tilde{\mathbf{p}}(t)]\tilde{\mathbf{p}}(t)\right\|}, \tag{A-4}$$

where $\|\mathbf{a}\|$ indicates the 1-norm of the vector $\mathbf{a}$, defined as the sum of the absolute values of the entries of the vector $\mathbf{a}$. Equilibrium solutions, denoted by $\hat{\mathbf{p}}$, satisfy

$$\hat{\mathbf{p}} = \frac{\tilde{\mathbf{A}}[\hat{\mathbf{p}}]\hat{\mathbf{p}}}{\mathbf{1}^{\mathsf{T}}_{2\omega g}\tilde{\mathbf{A}}[\hat{\mathbf{p}}]\hat{\mathbf{p}}}, \tag{A-5}$$

where the one norm can be replaced by $\mathbf{1}^{\mathsf{T}}_{2\omega g}\tilde{\mathbf{A}}[\hat{\mathbf{p}}]\hat{\mathbf{p}}$ because $\hat{\mathbf{p}}$ is nonnegative.

A.2.1 Linearization at the boundary equilibria

In this section[2] we derive the linear approximation to the dynamics in the neighborhood of a homozgote boundary equilibrium. The stability of a such an equilibrium to invasions by the other allele is determined by the magnitude of the largest eigenvalue of the Jacobian matrix of the frequency model evaluated at the equilibrium. If the magnitude of this eigenvalue is larger than one, then the equilibrium is unstable. The Jacobian matrix,

$$\mathbf{M} = \left.\frac{\mathrm{d}\tilde{\mathbf{p}}(t+1)}{\mathrm{d}\tilde{\mathbf{p}}(t)}\right|_{\hat{\mathbf{p}}}, \tag{A-6}$$

is obtained by differentiating equation (A-4) and evaluating the resulting derivatives at the boundary equilibrium. This requires a long series of matrix calculus operations, and repeatedly takes advantage of the fact that $\hat{\mathbf{p}}$ at the boundary contains zeros for the blocks corresponding to Aa and aa genotypes.

For notational convenience, first define a matrix $\mathbf{B}$ as

$$\mathbf{B}[\tilde{\mathbf{p}}] = \frac{\tilde{\mathbf{A}}[\tilde{\mathbf{p}}]}{\mathbf{1}^{\mathsf{T}}_{2\omega g}\tilde{\mathbf{A}}[\tilde{\mathbf{p}}]\tilde{\mathbf{p}}}, \tag{A-7}$$

such that

$$\tilde{\mathbf{p}}(t+1) = \mathbf{B}[\tilde{\mathbf{p}}(t)]\tilde{\mathbf{p}}(t). \tag{A-8}$$

Differentiate equation (A-8) to obtain

$$\mathrm{d}\tilde{\mathbf{p}}(t+1) = \mathbf{B}\mathrm{d}\tilde{\mathbf{p}}(t) + \left(\mathrm{d}\tilde{\mathbf{B}}\right)\tilde{\mathbf{p}}(t), \tag{A-9}$$

where the explicit dependence of $\mathbf{B}$ on $\tilde{\mathbf{p}}$ has been omitted to avoid a cluttering of brackets. Multiply the second term by an $2\omega g \times 2\omega g$ identity matrix,

$$\mathrm{d}\tilde{\mathbf{p}}(t+1) = \mathbf{B}\mathrm{d}\tilde{\mathbf{p}}(t) + \mathbf{I}_{2\omega g}\left(\mathrm{d}\mathbf{B}\right)\tilde{\mathbf{p}}(t). \tag{A-10}$$

and apply the vec operator to both sides, remembering that as $\tilde{\mathbf{p}}$ is a vector, $\mathrm{vec}\tilde{\mathbf{p}} = \tilde{\mathbf{p}}$,

$$\mathrm{d}\tilde{\mathbf{p}}(t+1) = \mathbf{B}\mathrm{d}\tilde{\mathbf{p}}(t) + \mathrm{vec}\left[\mathbf{I}_{2\omega g}\left(\mathrm{d}\mathbf{B}\right)\tilde{\mathbf{p}}(t)\right]. \tag{A-11}$$

Next apply Roth's theorem (Roth, 1934), $\mathrm{vec}\mathbf{ABC} = \left(\mathbf{C}^{\mathsf{T}} \otimes \mathbf{A}\right)\mathrm{vec}\mathbf{B}$, to replace the vec operator with the Kronecker product:

$$\mathrm{d}\tilde{\mathbf{p}}(t+1) = \mathbf{B}\mathrm{d}\tilde{\mathbf{p}}(t) + \left(\tilde{\mathbf{p}}^{\mathsf{T}}(t) \otimes \mathbf{I}_{2\omega g}\right)\mathrm{dvec}\mathbf{B}. \tag{A-12}$$

[2] This section is modified from Appendix B of de Vries and Caswell (2019) under the terms of a Creative Commons BY-NC license. The derivation here is modified to account for the presence of the two sexes.

Then the first identification theorem and the chain rule together give the following formula for the Jacobian (Magnus and Neudecker, 1985; Caswell, 2007),

$$\mathbf{M} = \left.\frac{\mathrm{d}\tilde{\mathbf{p}}(t+1)}{\mathrm{d}\tilde{\mathbf{p}}(t)}\right|_{\hat{\mathbf{p}}}, \tag{A-13}$$

$$= \mathbf{B}[\hat{\mathbf{p}}] + (\hat{\mathbf{p}}^{\mathsf{T}} \otimes \mathbf{I}_{2\omega g})\left.\frac{\partial \mathrm{vec}\mathbf{B}[\tilde{\mathbf{p}}]}{\partial \tilde{\mathbf{p}}^{\mathsf{T}}}\right|_{\hat{\mathbf{p}}}. \tag{A-14}$$

Our aim is to express the Jacobian matrix $\mathbf{M}$ in terms of the genotype specific matrices, $\mathbf{U}_i$, $\mathbf{F}_i$, $\mathbf{U}'_i$ and $\mathbf{F}'_i$. We choose to analyze the Jacobian at the AA boundary; the expression at the aa boundary can be derived afterwards using symmetry arguments. First it will be convenient to define the scalar $f(\tilde{\mathbf{p}})$ as

$$f(\tilde{\mathbf{p}}) = \frac{1}{\mathbf{1}^{\mathsf{T}}_{2\omega g}\tilde{\mathbf{A}}[\tilde{\mathbf{p}}]\tilde{\mathbf{p}}}, \tag{A-15}$$

so that

$$\mathbf{B}[\tilde{\mathbf{p}}] = f(\tilde{\mathbf{p}})\tilde{\mathbf{A}}[\tilde{\mathbf{p}}]. \tag{A-16}$$

Where it does not create confusion, we will drop the explicit dependence of $\tilde{\mathbf{A}}$, $\mathbf{B}$, and f on $\tilde{\mathbf{p}}$. Take the vec of both sides of equation (A-16) and differentiate to obtain

$$\mathrm{dvec}\mathbf{B} = \mathrm{vec}\tilde{\mathbf{A}}\mathrm{d}f + f\mathrm{dvec}\tilde{\mathbf{A}}, \tag{A-17}$$

or

$$\frac{\partial \mathrm{vec}\mathbf{B}}{\partial \tilde{\mathbf{p}}^{\mathsf{T}}} = \mathrm{vec}\tilde{\mathbf{A}}\frac{\partial f}{\partial \tilde{\mathbf{p}}^{\mathsf{T}}} + f(\tilde{\mathbf{p}})\frac{\partial \mathrm{vec}\tilde{\mathbf{A}}}{\partial \tilde{\mathbf{p}}^{\mathsf{T}}}. \tag{A-18}$$

Next differentiate f in equation (A-15) to obtain

$$\mathrm{d}f = \frac{-1}{\left(\mathbf{1}^{\mathsf{T}}_{2\omega g}\tilde{\mathbf{A}}[\tilde{\mathbf{p}}]\tilde{\mathbf{p}}\right)^2}\left[\mathbf{1}^{\mathsf{T}}_{2\omega g}\left(\mathrm{d}\tilde{\mathbf{A}}\right)\tilde{\mathbf{p}} + \mathbf{1}^{\mathsf{T}}_{2\omega g}\tilde{\mathbf{A}}\mathrm{d}\tilde{\mathbf{p}}\right]. \tag{A-19}$$

All the terms in the Jacobian are evaluated at the AA boundary, which simplifies some of the equations, e.g., $\tilde{\mathbf{A}}[\hat{\mathbf{p}}]\hat{\mathbf{p}} = \lambda_{AA}\hat{\mathbf{p}}$, and therefore

$$\mathbf{1}^{\mathsf{T}}_{2\omega g}\tilde{\mathbf{A}}[\hat{\mathbf{p}}]\hat{\mathbf{p}} = \lambda_{AA}. \tag{A-20}$$

Evaluate the differential of f at the boundary and use equation (A-20) to obtain

$$\mathrm{d}f(\hat{\mathbf{p}}) = \frac{-1}{\lambda^2_{AA}}\left.\left[\mathbf{1}^{\mathsf{T}}_{2\omega g}\left(\mathrm{d}\tilde{\mathbf{A}}\right)\hat{\mathbf{p}} + \mathbf{1}^{\mathsf{T}}_{2\omega g}\tilde{\mathbf{A}}\mathrm{d}\tilde{\mathbf{p}}\right]\right|_{\hat{\mathbf{p}}}. \tag{A-21}$$

The first term in this sum, $\mathbf{1}^{\mathsf{T}}_{2\omega g}\left(\mathrm{d}\tilde{\mathbf{A}}\right)\hat{\mathbf{p}}$, is equal to zero because

$$q'_a + q'_A = 1, \tag{A-22}$$

$$\mathrm{d}q'_a + \mathrm{d}q'_A = 0, \tag{A-23}$$

and therefore every column in $\left(\mathrm{d}\tilde{\mathbf{A}}\right)$ sums to zero, see equation A-3.

Substituting equation (A-21) into equation (A-17) and evaluating at the boundary yields

$$\mathrm{dvec}\mathbf{B} = \frac{-1}{\lambda^2_{AA}}\mathrm{vec}\tilde{\mathbf{A}}\left[\mathbf{1}^{\mathsf{T}}_{2\omega g}\tilde{\mathbf{A}}\mathrm{d}\tilde{\mathbf{p}}\right] + \frac{1}{\lambda_{AA}}\mathrm{dvec}\tilde{\mathbf{A}}, \tag{A-24}$$

or

$$\left.\frac{\partial \mathrm{vec}\mathbf{B}}{\partial \tilde{\mathbf{p}}^{\mathsf{T}}}\right|_{\hat{\mathbf{p}}} = \frac{-1}{\lambda_{AA}^2}\left(\mathrm{vec}\tilde{\mathbf{A}}\right)\left(\mathbf{1}_{2\omega g}^{\mathsf{T}}\tilde{\mathbf{A}}\right) + \frac{1}{\lambda_{AA}}\left.\frac{\partial \mathrm{vec}\tilde{\mathbf{A}}}{\partial \tilde{\mathbf{p}}^{\mathsf{T}}}\right|_{\hat{\mathbf{p}}}. \tag{A-25}$$

Finally substituting the expression above into equation A-14 yields the Jacobian matrix:

$$\mathbf{M} = \mathbf{B}[\hat{\mathbf{p}}] + (\hat{\mathbf{p}}^{\mathsf{T}} \otimes \mathbf{I}_{2\omega g})\left.\frac{\partial \mathrm{vec}\mathbf{B}}{\partial \tilde{\mathbf{p}}^{\mathsf{T}}}\right|_{\hat{\mathbf{p}}}, \tag{A-26}$$

$$= \underbrace{\mathbf{B}[\hat{\mathbf{p}}]}_{\text{(A)}} \underbrace{- \frac{1}{\lambda_{AA}^2}(\hat{\mathbf{p}}^{\mathsf{T}} \otimes \mathbf{I}_{2\omega g})\left(\mathrm{vec}\tilde{\mathbf{A}}\right)\left(\mathbf{1}_{2\omega g}^{\mathsf{T}}\tilde{\mathbf{A}}\right)}_{\text{(B)}}$$

$$+ \underbrace{\frac{1}{\lambda_{AA}}(\hat{\mathbf{p}}^{\mathsf{T}} \otimes \mathbf{I}_{2\omega g})\left.\frac{\partial \mathrm{vec}\tilde{\mathbf{A}}}{\partial \tilde{\mathbf{p}}^{\mathsf{T}}}\right|_{\hat{\mathbf{p}}}}_{\text{(C)}}, \tag{A-27}$$

where we have identified the three terms as (A), (B), and (C).

A.2.2 Components of the Jacobian

The next task is to work out all the terms in the above expression for the Jacobian. We start with (A),

$$\mathbf{B}[\hat{\mathbf{p}}] = \frac{\tilde{\mathbf{A}}[\hat{\mathbf{p}}]}{\mathbf{1}_{\omega g}^{\mathsf{T}}\tilde{\mathbf{A}}[\hat{\mathbf{p}}]\hat{\mathbf{p}}} \tag{A-28}$$

$$= \frac{1}{\lambda_{AA}}\left(\begin{array}{cc|cc|cc}
\mathbf{U}_{AA} + \alpha\mathbf{F}_{AA} & \mathbf{0} & \frac{1}{2}\alpha\mathbf{F}_{Aa} & \mathbf{0} & \mathbf{0} & \mathbf{0} \\
(1-\alpha)\mathbf{F}_{AA} & \mathbf{U}'_{AA} & \frac{1}{2}(1-\alpha)\mathbf{F}_{Aa} & \mathbf{0} & \mathbf{0} & \mathbf{0} \\
\hline
\mathbf{0} & \mathbf{0} & \mathbf{U}_{Aa} + \frac{1}{2}\alpha\mathbf{F}_{Aa} & \mathbf{0} & \alpha\mathbf{F}_{aa} & \mathbf{0} \\
\mathbf{0} & \mathbf{0} & \frac{1}{2}(1-\alpha)\mathbf{F}_{Aa} & \mathbf{U}'_{Aa} & (1-\alpha)\mathbf{F}_{aa} & \mathbf{0} \\
\hline
\mathbf{0} & \mathbf{0} & \mathbf{0} & \mathbf{0} & \mathbf{U}_{aa} & \mathbf{0} \\
\mathbf{0} & \mathbf{0} & \mathbf{0} & \mathbf{0} & \mathbf{0} & \mathbf{U}'_{aa}
\end{array}\right). \tag{A-29}$$

Next we turn our attention to the second term, (B),

$$\text{(B)} = -\frac{1}{\lambda_{AA}^2}(\hat{\mathbf{p}}^{\mathsf{T}} \otimes \mathbf{I}_{2\omega g})\left(\mathrm{vec}\tilde{\mathbf{A}}\right)\left(\mathbf{1}_{2\omega g}^{\mathsf{T}}\tilde{\mathbf{A}}\right). \tag{A-30}$$

Using Roth's theorem, $(\mathbf{C}^{\mathsf{T}} \otimes \mathbf{A})\,\mathrm{vec}\mathbf{B} = \mathrm{vec}\mathbf{ABC}$, we can simplify as follows

$$(\hat{\mathbf{p}}^{\mathsf{T}} \otimes \mathbf{I}_{2\omega g})\,\mathrm{vec}\left(\tilde{\mathbf{A}}\,[\hat{\mathbf{p}}]\right) = \mathrm{vec}\left(\mathbf{I}_{2\omega g}\tilde{\mathbf{A}}[\hat{\mathbf{p}}]\hat{\mathbf{p}}\right)$$

$$= \lambda_{AA}\hat{\mathbf{p}}, \tag{A-31}$$

which yields

$$\text{(B)} = -\frac{1}{\lambda_{AA}}\hat{\mathbf{p}}\left(\mathbf{1}_{\omega g}^{\mathsf{T}}\tilde{\mathbf{A}}\,[\hat{\mathbf{p}}]\right). \tag{A-32}$$

Substitute the population vector analyzed at the AA boundary,

$$\hat{\mathbf{p}} = \left(\begin{array}{c}
\hat{\mathbf{p}}_{AA} \\
\hat{\mathbf{p}}'_{AA} \\
\hline
0 \\
0 \\
\hline
0 \\
0
\end{array}\right). \tag{A-33}$$

into equation (A-32) and write the result in terms of the block matrices to obtain

$$
-\frac{1}{\lambda_{AA}}\left(\begin{array}{cc|cc|cc}
\hat{\mathbf{p}}_{AA}\otimes\mathbf{1}_\omega^\mathsf{T}\mathbf{U}_{AA} & \hat{\mathbf{p}}_{AA}\otimes\mathbf{1}_\omega^\mathsf{T}\mathbf{U}'_{AA} & \hat{\mathbf{p}}_{AA}\otimes\mathbf{1}_\omega^\mathsf{T}\mathbf{U}_{Aa} & \hat{\mathbf{p}}_{AA}\otimes\mathbf{1}_\omega^\mathsf{T}\mathbf{U}'_{Aa} & \hat{\mathbf{p}}_{AA}\otimes\mathbf{1}_\omega^\mathsf{T}\mathbf{U}_{aa} & \hat{\mathbf{p}}_{AA}\otimes\mathbf{1}_\omega^\mathsf{T}\mathbf{U}'_{aa} \\
\hat{\mathbf{p}}'_{AA}\otimes\mathbf{1}_\omega^\mathsf{T}\mathbf{U}_{AA} & \hat{\mathbf{p}}'_{AA}\otimes\mathbf{1}_\omega^\mathsf{T}\mathbf{U}'_{AA} & \hat{\mathbf{p}}'_{AA}\otimes\mathbf{1}_\omega^\mathsf{T}\mathbf{U}_{Aa} & \hat{\mathbf{p}}'_{AA}\otimes\mathbf{1}_\omega^\mathsf{T}\mathbf{U}'_{Aa} & \hat{\mathbf{p}}'_{AA}\otimes\mathbf{1}_\omega^\mathsf{T}\mathbf{U}_{aa} & \hat{\mathbf{p}}'_{AA}\otimes\mathbf{1}_\omega^\mathsf{T}\mathbf{U}'_{aa} \\ \hline
\mathbf{0} & \mathbf{0} & \mathbf{0} & \mathbf{0} & \mathbf{0} & \mathbf{0} \\
\mathbf{0} & \mathbf{0} & \mathbf{0} & \mathbf{0} & \mathbf{0} & \mathbf{0} \\ \hline
\mathbf{0} & \mathbf{0} & \mathbf{0} & \mathbf{0} & \mathbf{0} & \mathbf{0} \\
\mathbf{0} & \mathbf{0} & \mathbf{0} & \mathbf{0} & \mathbf{0} & \mathbf{0}
\end{array}\right)
-\frac{1}{\lambda_{AA}}\left(\begin{array}{cc|cc|cc}
\alpha\hat{\mathbf{p}}_{AA}\otimes\mathbf{1}_\omega^\mathsf{T}\mathbf{F}_{AA} & \mathbf{0} & \alpha\hat{\mathbf{p}}_{AA}\otimes\mathbf{1}_\omega^\mathsf{T}\mathbf{F}_{Aa} & \mathbf{0} & \alpha\hat{\mathbf{p}}_{AA}\otimes\mathbf{1}_\omega^\mathsf{T}\mathbf{F}_{aa} & \mathbf{0} \\
(1-\alpha)\hat{\mathbf{p}}'_{AA}\otimes\mathbf{1}_\omega^\mathsf{T}\mathbf{F}_{AA} & \mathbf{0} & (1-\alpha)\hat{\mathbf{p}}'_{AA}\otimes\mathbf{1}_\omega^\mathsf{T}\mathbf{F}_{Aa} & \mathbf{0} & (1-\alpha)\hat{\mathbf{p}}'_{AA}\otimes\mathbf{1}_\omega^\mathsf{T}\mathbf{F}_{aa} & \mathbf{0} \\ \hline
\mathbf{0} & \mathbf{0} & \mathbf{0} & \mathbf{0} & \mathbf{0} & \mathbf{0} \\
\mathbf{0} & \mathbf{0} & \mathbf{0} & \mathbf{0} & \mathbf{0} & \mathbf{0} \\ \hline
\mathbf{0} & \mathbf{0} & \mathbf{0} & \mathbf{0} & \mathbf{0} & \mathbf{0} \\
\mathbf{0} & \mathbf{0} & \mathbf{0} & \mathbf{0} & \mathbf{0} & \mathbf{0}
\end{array}\right)
\tag{A-34}
$$

To derive term (C) in the Jacobian, we first derive a useful expression for $\mathrm{vec}\tilde{\mathbf{A}}$ in terms of its component block matrices. The matrix $\tilde{\mathbf{A}}$ can be decomposed into 36 $\omega \times \omega$ block matrices, as in equation (A-3), so that for example

$$\mathbf{A}_{11} = \mathbf{U}_{AA} + q'_A\alpha\mathbf{F}_{AA}, \tag{A-35}$$
$$\mathbf{A}_{22} = \mathbf{U}'_{AA}, \tag{A-36}$$

and

$$\mathbf{A}_{21} = (1-\alpha)q'_A\mathbf{F}_{AA}, \tag{A-37}$$
$$\mathbf{A}_{12} = \mathbf{0}. \tag{A-38}$$

The matrix $\tilde{\mathbf{A}}$ can then be written as

$$\tilde{\mathbf{A}} = \sum_{i,j=1}^{6} \mathbf{E}_{ij}\otimes\mathbf{A}_{ij}, \tag{A-39}$$
$$= \sum_{i,j=1}^{6} \left(\mathbf{e}_i\mathbf{e}_j^\mathsf{T}\right)\otimes\left(\mathbf{A}_{ij}\mathbf{I}_\omega\right), \tag{A-40}$$

where we have used the definition of the matrix $\mathbf{E}_{ij} = \mathbf{e}_i\mathbf{e}_j^\mathsf{T}$. Using $\mathbf{AC}\otimes\mathbf{BD} = (\mathbf{A}\otimes\mathbf{B})(\mathbf{C}\otimes\mathbf{D})$, equation (A-40) can be rewritten as

$$\tilde{\mathbf{A}} = \sum_{i,j=1}^{6} \left(\mathbf{e}_i\otimes\mathbf{A}_{ij}\right)\left(\mathbf{e}_j^\mathsf{T}\otimes\mathbf{I}_\omega\right), \tag{A-41}$$

next use the identity $\sum_i \left(\mathbf{e}_i\otimes\mathbf{I}_\omega\right)\mathbf{A}_{ij} = \sum_i \mathbf{e}_i\otimes\mathbf{A}_{ij}$ to rewrite this again

$$\tilde{\mathbf{A}} = \sum_{i,j=1}^{6} \left(\mathbf{e}_i\otimes\mathbf{I}_\omega\right)\mathbf{A}_{ij}\left(\mathbf{e}_j^\mathsf{T}\otimes\mathbf{I}_\omega\right). \tag{A-42}$$

Use Roth's theorem, $\mathrm{vec}\mathbf{ABC} = \left(\mathbf{C}^\mathsf{T}\otimes\mathbf{A}\right)\mathrm{vec}\mathbf{B}$, where $\mathbf{A} = (\mathbf{e}_i\otimes\mathbf{I}_\omega)$, $\mathbf{B} = \mathbf{A}_{ij}$ and $\mathbf{C} = \left(\mathbf{e}_j^\mathsf{T}\otimes\mathbf{I}_\omega\right)$, to obtain the following formula for $\mathrm{vec}\mathbf{A}$:

$$\mathrm{vec}\tilde{\mathbf{A}} = \sum_{i,j}^{6} \left(\mathbf{e}_j\otimes\mathbf{I}_\omega\right)\otimes\left(\mathbf{e}_i\otimes\mathbf{I}_\omega\right)\mathrm{vec}\mathbf{A}_{ij}. \tag{A-43}$$

Armed with this expression for $\text{vec}\tilde{\mathbf{A}}$, we analyze term Ⓒ in the Jacobian. Replace the derivative of $\text{vec}\tilde{\mathbf{A}}$ with equation A-43, such that

$$\frac{1}{\lambda_{AA}}(\hat{\mathbf{p}}^{\mathsf{T}} \otimes \mathbf{I}_{2\omega g})\frac{\partial \text{vec}\mathbf{A}}{\partial \tilde{\mathbf{p}}^{\mathsf{T}}}\bigg|_{\hat{\mathbf{p}}} = \frac{1}{\lambda_{AA}}\sum_{i,j=1}^{6}(\hat{\mathbf{p}}^{\mathsf{T}} \otimes \mathbf{I}_{2\omega g})\left((\mathbf{e}_j \otimes \mathbf{I}_\omega) \otimes (\mathbf{e}_i \otimes \mathbf{I}_\omega)\right)\frac{\partial \text{vec}\mathbf{A}_{ij}}{\partial \tilde{\mathbf{p}}^{\mathsf{T}}}\bigg|_{\hat{\mathbf{p}}}. \tag{A-44}$$

Use $(\mathbf{A} \otimes \mathbf{B})(\mathbf{C} \otimes \mathbf{D}) = \mathbf{AC} \otimes \mathbf{BD}$ to rewrite

$$(\hat{\mathbf{p}}^{\mathsf{T}} \otimes \mathbf{I}_{2\omega g})\left((\mathbf{e}_j \otimes \mathbf{I}_\omega) \otimes (\mathbf{e}_i \otimes \mathbf{I}_\omega)\right) = (\hat{\mathbf{p}}^{\mathsf{T}}(\mathbf{e}_j \otimes \mathbf{I}_\omega)) \otimes (\mathbf{I}_{2\omega g}(\mathbf{e}_i \otimes \mathbf{I}_\omega)), \tag{A-45}$$

substituting this expression into the right hand side of equation (A-44) yields

$$\frac{1}{\lambda_{AA}}(\hat{\mathbf{p}}^{\mathsf{T}} \otimes \mathbf{I}_{2\omega g})\frac{\partial \text{vec}\mathbf{A}}{\partial \tilde{\mathbf{p}}^{\mathsf{T}}}\bigg|_{\hat{\mathbf{p}}} = \frac{1}{\lambda_{AA}}\sum_{i,j=1}^{6}(\hat{\mathbf{p}}^{\mathsf{T}}(\mathbf{e}_j \otimes \mathbf{I}_\omega)) \otimes (\mathbf{I}_{2\omega g}(\mathbf{e}_i \otimes \mathbf{I}_\omega))\frac{\partial \text{vec}\mathbf{A}_{ij}}{\partial \tilde{\mathbf{p}}^{\mathsf{T}}}\bigg|_{\hat{\mathbf{p}}}. \tag{A-46}$$

Substitute $\hat{\mathbf{p}}^{\mathsf{T}} = (\hat{\mathbf{p}}_{AA}^{\mathsf{T}}, \hat{\mathbf{p}}_{AA}'^{\mathsf{T}}, \mathbf{0}, \mathbf{0}, \mathbf{0}, \mathbf{0})$ into the right-hand side of equation (A-46), so that only terms with $j = 1$ and $j = 2$ are nonzero, yielding

$$\begin{aligned}\frac{1}{\lambda_{AA}}(\hat{\mathbf{p}}^{\mathsf{T}} \otimes \mathbf{I}_{2\omega g})\frac{\partial \text{vec}\mathbf{A}}{\partial \tilde{\mathbf{p}}^{\mathsf{T}}}\bigg|_{\hat{\mathbf{p}}} &= \frac{1}{\lambda_{AA}}\sum_{i=1}^{6}(\hat{\mathbf{p}}_{AA}^{\mathsf{T}} \otimes (\mathbf{e}_i \otimes \mathbf{I}_\omega))\frac{\partial \text{vec}\mathbf{A}_{i1}}{\partial \tilde{\mathbf{p}}^{\mathsf{T}}}\bigg|_{\hat{\mathbf{p}}} \\ &+ \frac{1}{\lambda_{AA}}\sum_{i=1}^{6}\left(\hat{\mathbf{p}}_{AA}'^{\mathsf{T}} \otimes (\mathbf{e}_i \otimes \mathbf{I}_\omega)\right)\frac{\partial \text{vec}\mathbf{A}_{i2}}{\partial \tilde{\mathbf{p}}^{\mathsf{T}}}\bigg|_{\hat{\mathbf{p}}},\end{aligned} \tag{A-47}$$

Since none of the $\mathbf{A}_{i2}$ are a function of the frequency vector,

$$\frac{\partial \text{vec}\mathbf{A}_{i2}}{\partial \tilde{\mathbf{p}}^{\mathsf{T}}}\bigg|_{\hat{\mathbf{p}}} = 0, \text{ for all } i. \tag{A-48}$$

Next write down each term in the sum over i and take the derivative of the $\text{vec}\mathbf{A}_{i1}$'s to obtain

$$\begin{aligned}\frac{1}{\lambda_{AA}}(\hat{\mathbf{p}}^{\mathsf{T}} \otimes \mathbf{I}_{2\omega g})\frac{\partial \text{vec}\mathbf{A}}{\partial \tilde{\mathbf{p}}^{\mathsf{T}}}\bigg|_{\hat{\mathbf{p}}} &= \frac{\alpha}{\lambda_{AA}}\left[\hat{\mathbf{p}}_{AA}^{\mathsf{T}} \otimes (\mathbf{e}_1 \otimes \mathbf{I}_\omega) - \hat{\mathbf{p}}_{AA}^{\mathsf{T}} \otimes (\mathbf{e}_3 \otimes \mathbf{I}_\omega)\right]\text{vec}(\mathbf{F}_{AA})\frac{\partial q_A'}{\partial \tilde{\mathbf{p}}^{\mathsf{T}}}\bigg|_{\hat{\mathbf{p}}}, \\ &+ \frac{(1-\alpha)}{\lambda_{AA}}\left[\hat{\mathbf{p}}_{AA}^{\mathsf{T}} \otimes (\mathbf{e}_2 \otimes \mathbf{I}_\omega) - \hat{\mathbf{p}}_{AA}^{\mathsf{T}} \otimes (\mathbf{e}_4 \otimes \mathbf{I}_\omega)\right]\text{vec}(\mathbf{F}_{AA})\frac{\partial q_A'}{\partial \tilde{\mathbf{p}}^{\mathsf{T}}}\bigg|_{\hat{\mathbf{p}}},\end{aligned}$$

Finally apply Roth's theorem (Roth, 1934), $(\mathbf{C}^{\mathsf{T}} \otimes \mathbf{A})\,\text{vec}\mathbf{B} = \text{vec}\mathbf{ABC}$, to the equation above (e.g. $\mathbf{C}^{\mathsf{T}} = \hat{\mathbf{p}}_{AA}^{\mathsf{T}}$, $\mathbf{A} = (\mathbf{e}_1 \otimes \mathbf{I}_\omega)$, and $\text{vec}\mathbf{B} = \text{vec}\mathbf{F}_{AA}$) to write this as

$$\begin{aligned}\frac{1}{\lambda_{AA}}(\hat{\mathbf{p}}^{\mathsf{T}} \otimes \mathbf{I}_{\omega g})\frac{\partial \text{vec}\mathbf{A}}{\partial \tilde{\mathbf{p}}^{\mathsf{T}}}\bigg|_{\hat{\mathbf{p}}} &= \frac{\alpha}{\lambda_{AA}}\left[\text{vec}((\mathbf{e}_1 \otimes \mathbf{I}_\omega)\mathbf{F}_{AA}\hat{\mathbf{p}}_{AA}) - \text{vec}((\mathbf{e}_3 \otimes \mathbf{I}_\omega)\mathbf{F}_{AA}\hat{\mathbf{p}}_{AA})\right]\frac{\partial q_A'}{\partial \tilde{\mathbf{p}}^{\mathsf{T}}}\bigg|_{\hat{\mathbf{p}}} \\ &+ \frac{(1-\alpha)}{\lambda_{AA}}\left[\text{vec}((\mathbf{e}_2 \otimes \mathbf{I}_\omega)\mathbf{F}_{AA}\hat{\mathbf{p}}_{AA}) - \text{vec}((\mathbf{e}_4 \otimes \mathbf{I}_\omega)\mathbf{F}_{AA}\hat{\mathbf{p}}_{AA})\right]\frac{\partial q_A'}{\partial \tilde{\mathbf{p}}^{\mathsf{T}}}\bigg|_{\hat{\mathbf{p}}}.\end{aligned}$$

Written in terms of block matrices this expression yields

$$\frac{1}{\lambda_{AA}}\left(\begin{array}{cc|cc|cc} \mathbf{0} & \alpha(\mathbf{F}_{AA}\hat{\mathbf{p}}_{AA})\otimes\frac{\partial q'_A}{\partial \mathbf{p}'^{\mathsf{T}}_{AA}} & \mathbf{0} & \alpha(\mathbf{F}_{AA}\hat{\mathbf{p}}_{AA})\otimes\frac{\partial q'_A}{\partial \mathbf{p}'^{\mathsf{T}}_{Aa}} & \mathbf{0} & \alpha(\mathbf{F}_{AA}\hat{\mathbf{p}}_{AA})\otimes\frac{\partial q'_A}{\partial \mathbf{p}'^{\mathsf{T}}_{aa}} \\ \mathbf{0} & (1-\alpha)(\mathbf{F}_{AA}\hat{\mathbf{p}}_{AA})\otimes\frac{\partial q'_A}{\partial \mathbf{p}'^{\mathsf{T}}_{AA}} & \mathbf{0} & (1-\alpha)(\mathbf{F}_{AA}\hat{\mathbf{p}}_{AA})\otimes\frac{\partial q'_A}{\partial \mathbf{p}'^{\mathsf{T}}_{Aa}} & \mathbf{0} & (1-\alpha)(\mathbf{F}_{AA}\hat{\mathbf{p}}_{AA})\otimes\frac{\partial q'_A}{\partial \mathbf{p}'^{\mathsf{T}}_{aa}} \\ \hline \mathbf{0} & -\alpha(\mathbf{F}_{AA}\hat{\mathbf{p}}_{AA})\otimes\frac{\partial q'_A}{\partial \mathbf{p}'^{\mathsf{T}}_{AA}} & \mathbf{0} & -\alpha(\mathbf{F}_{AA}\hat{\mathbf{p}}_{AA})\otimes\frac{\partial q'_A}{\partial \mathbf{p}'^{\mathsf{T}}_{Aa}} & \mathbf{0} & -\alpha(\mathbf{F}_{AA}\hat{\mathbf{p}}_{AA})\otimes\frac{\partial q'_A}{\partial \mathbf{p}'^{\mathsf{T}}_{aa}} \\ \mathbf{0} & -(1-\alpha)(\mathbf{F}_{AA}\hat{\mathbf{p}}_{AA})\otimes\frac{\partial q'_A}{\partial \mathbf{p}'^{\mathsf{T}}_{AA}} & \mathbf{0} & -(1-\alpha)(\mathbf{F}_{AA}\hat{\mathbf{p}}_{AA})\otimes\frac{\partial q'_A}{\partial \mathbf{p}'^{\mathsf{T}}_{Aa}} & \mathbf{0} & -(1-\alpha)(\mathbf{F}_{AA}\hat{\mathbf{p}}_{AA})\otimes\frac{\partial q'_A}{\partial \mathbf{p}'^{\mathsf{T}}_{aa}} \\ \hline \mathbf{0} & \mathbf{0} & \mathbf{0} & \mathbf{0} & \mathbf{0} & \mathbf{0} \\ \mathbf{0} & \mathbf{0} & \mathbf{0} & \mathbf{0} & \mathbf{0} & \mathbf{0} \end{array}\right). \tag{A-49}$$

Equation (A-49) requires the derivative of the frequency of allele A in the gamete pool with respect to the population frequency vector:

$$\left.\frac{\partial q'_A}{\partial \tilde{\mathbf{p}}^{\mathsf{T}}}\right|_{\hat{\mathbf{p}}}. \tag{A-50}$$

Start with equation (5) from the main text:

$$\begin{pmatrix} q'_A \\ q'_a \end{pmatrix} = \frac{\mathbf{W}'\mathbf{F}'\mathbf{n}'}{\|\mathbf{W}'\mathbf{F}'\mathbf{n}'\|}, \tag{A-51}$$

therefore

$$q'_A = \frac{\mathbf{e}_1^{\mathsf{T}}\mathbf{W}'\mathbf{F}'\mathbf{p}'}{\mathbf{1}_2^{\mathsf{T}}\mathbf{W}'\mathbf{F}'\mathbf{p}'}, \tag{A-52}$$

where we can substitute $\mathbf{p}'$ for $\mathbf{n}'$ because of homogeneity and where the one norm can be replaced by $\mathbf{1}_2^{\mathsf{T}}\mathbf{W}'\mathbf{F}'\mathbf{p}'$ because $\mathbf{p}'$ is nonnegative. For convenience, we will denote the normalizing factor in the denominator with $\mathrm{p_n}$,

$$\mathrm{p_n} = \mathbf{1}_2^{\mathsf{T}}\mathbf{W}'\mathbf{F}'\mathbf{p}' \tag{A-53}$$

Taking the derivative of q'_A yields

$$\frac{\partial q'_A}{\partial \tilde{\mathbf{p}}^{\mathsf{T}}} = \frac{1}{\mathrm{p_n}}\mathbf{e}_1^{\mathsf{T}}\mathbf{W}'\mathbf{F}'\frac{\partial \mathbf{p}'}{\partial \tilde{\mathbf{p}}^{\mathsf{T}}} - \frac{\mathbf{e}_1^{\mathsf{T}}\mathbf{W}'\mathbf{F}'\mathbf{p}'}{\mathrm{p_n^2}}\left(\mathbf{1}_2^{\mathsf{T}}\mathbf{W}'\mathbf{F}'\frac{\partial \mathbf{p}'}{\partial \tilde{\mathbf{p}}^{\mathsf{T}}}\right). \tag{A-54}$$

Recall

$$\mathbf{p}' = \begin{pmatrix} \mathbf{p}'_{AA} \\ \mathbf{p}'_{Aa} \\ \mathbf{p}'_{aa} \end{pmatrix}, \tag{A-55}$$

and

$$\tilde{\mathbf{p}} = \left(\begin{array}{c} \mathbf{p}_{AA} \\ \mathbf{p}'_{AA} \\ \hline \mathbf{p}_{Aa} \\ \mathbf{p}'_{Aa} \\ \hline \mathbf{p}_{aa} \\ \mathbf{p}'_{aa} \end{array}\right), \tag{A-56}$$

to calculate

$$\frac{\partial \mathbf{p}'}{\partial \tilde{\mathbf{p}}^{\mathsf{T}}} = \begin{pmatrix} \mathbf{0} & \mathbf{I} & \mathbf{0} & \mathbf{0} & \mathbf{0} & \mathbf{0} \\ \mathbf{0} & \mathbf{0} & \mathbf{0} & \mathbf{I} & \mathbf{0} & \mathbf{0} \\ \mathbf{0} & \mathbf{0} & \mathbf{0} & \mathbf{0} & \mathbf{0} & \mathbf{I} \end{pmatrix}. \tag{A-57}$$

First we will evaluate the first term in the sum in equation (A-54),

$$\begin{aligned}
\frac{1}{\mathrm{p_n}}\mathbf{e}_1^\mathsf{T}\mathbf{W}'\mathbf{F}'\frac{\partial\mathbf{p}'}{\partial\tilde{\mathbf{p}}^\mathsf{T}} &= \frac{1}{\mathrm{p_n}}(1,0)\begin{pmatrix} \mathbf{1}_\omega^\mathsf{T}\mathbf{F}'_{AA} & \frac{1}{2}\mathbf{1}_\omega^\mathsf{T}\mathbf{F}'_{Aa} & \mathbf{0} \\ \mathbf{0} & \frac{1}{2}\mathbf{1}_\omega^\mathsf{T}\mathbf{F}'_{Aa} & \mathbf{1}_\omega^\mathsf{T}\mathbf{F}'_{aa} \end{pmatrix}\begin{pmatrix} \mathbf{0} & \mathbf{I} & \mathbf{0} & \mathbf{0} & \mathbf{0} & \mathbf{0} \\ \mathbf{0} & \mathbf{0} & \mathbf{0} & \mathbf{I} & \mathbf{0} & \mathbf{0} \\ \mathbf{0} & \mathbf{0} & \mathbf{0} & \mathbf{0} & \mathbf{0} & \mathbf{I} \end{pmatrix} \\
&= \frac{1}{\mathrm{p_n}}\left(\mathbf{0}, \mathbf{1}_\omega^\mathsf{T}\mathbf{F}'_{AA}, \mathbf{0}, \frac{1}{2}\mathbf{1}_\omega^\mathsf{T}\mathbf{F}'_{Aa}, \mathbf{0}, \mathbf{0}\right) \qquad \text{(A-58)}
\end{aligned}$$

Similarly for the second term in the sum in equation (A-54),

$$\begin{aligned}
-\frac{\mathbf{e}_1^\mathsf{T}\mathbf{W}'\mathbf{F}'\mathbf{p}'}{\mathrm{p_n^2}}\left(\mathbf{1}_2^\mathsf{T}\mathbf{W}'\mathbf{F}'\frac{\partial\mathbf{p}'}{\partial\tilde{\mathbf{p}}^\mathsf{T}}\right) &= -\frac{1}{\mathrm{p_n}}(1,1)\begin{pmatrix} \mathbf{1}_\omega^\mathsf{T}\mathbf{F}'_{AA} & \frac{1}{2}\mathbf{1}_\omega^\mathsf{T}\mathbf{F}'_{Aa} & \mathbf{0} \\ \mathbf{0} & \frac{1}{2}\mathbf{1}_\omega^\mathsf{T}\mathbf{F}'_{Aa} & \mathbf{1}_\omega^\mathsf{T}\mathbf{F}'_{aa} \end{pmatrix}\begin{pmatrix} \mathbf{0} & \mathbf{I} & \mathbf{0} & \mathbf{0} & \mathbf{0} & \mathbf{0} \\ \mathbf{0} & \mathbf{0} & \mathbf{0} & \mathbf{I} & \mathbf{0} & \mathbf{0} \\ \mathbf{0} & \mathbf{0} & \mathbf{0} & \mathbf{0} & \mathbf{0} & \mathbf{I} \end{pmatrix} \\
&= -\frac{1}{\mathrm{p_n}}\left(\mathbf{0}, \mathbf{1}_\omega^\mathsf{T}\mathbf{F}'_{AA}, \mathbf{0}, \mathbf{1}_\omega^\mathsf{T}\mathbf{F}'_{Aa}, \mathbf{0}, \mathbf{1}_\omega^\mathsf{T}\mathbf{F}'_{aa}\right) \qquad \text{(A-59)}
\end{aligned}$$

Finally add equations (A-58) and (A-59) to obtain

$$\left.\frac{\partial q'_A}{\partial\tilde{\mathbf{p}}^\mathsf{T}}\right|_{\hat{\mathbf{p}}} = \frac{1}{\mathrm{p_n}}\left(\mathbf{0}, \mathbf{0}, \mathbf{0}, -\frac{1}{2}\mathbf{1}_\omega^\mathsf{T}\mathbf{F}'_{Aa}, \mathbf{0}, -\mathbf{1}_\omega^\mathsf{T}\mathbf{F}'_{aa}\right), \qquad \text{(A-60)}$$

where at the boundary

$$\begin{aligned}
\mathrm{p_n} &= \mathbf{1}_\omega^\mathsf{T}\mathbf{F}'_{AA}\hat{\mathbf{p}}'_{AA}, \qquad \text{(A-61)} \\
\mathbf{e}_1^\mathsf{T}\mathbf{W}'\mathbf{F}'\mathbf{p}' &= \mathbf{1}_\omega^\mathsf{T}\mathbf{F}'_{AA}\hat{\mathbf{p}}'_{AA} = \mathrm{p_n} \qquad \text{(A-62)}
\end{aligned}$$

Finally, plugging equation (A-60) into (A-49) yields

$$\frac{1}{\lambda_{AA}}(\hat{\mathbf{p}}^\mathsf{T}\otimes\mathbf{I}_{2\omega g})\left.\frac{\partial\mathrm{vec}\mathbf{A}}{\partial\tilde{\mathbf{p}}^\mathsf{T}}\right|_{\hat{\mathbf{p}}} =$$

$$\frac{1}{\lambda_{AA}}\left(\begin{array}{cc|cc|cc}
\mathbf{0} & \mathbf{0} & \mathbf{0} & -\frac{\alpha}{2\mathrm{p_n}}(\mathbf{F}_{AA}\hat{\mathbf{p}}_{AA})\otimes\mathbf{1}_\omega^\mathsf{T}\mathbf{F}'_{Aa} & \mathbf{0} & -\frac{\alpha}{\mathrm{p_n}}(\mathbf{F}_{AA}\hat{\mathbf{p}}_{AA})\otimes\mathbf{1}_\omega^\mathsf{T}\mathbf{F}'_{aa} \\
\mathbf{0} & \mathbf{0} & \mathbf{0} & -\frac{(1-\alpha)}{2\mathrm{p_n}}(\mathbf{F}_{AA}\hat{\mathbf{p}}_{AA})\otimes\mathbf{1}_\omega^\mathsf{T}\mathbf{F}'_{Aa} & \mathbf{0} & -\frac{(1-\alpha)}{\mathrm{p_n}}(\mathbf{F}_{AA}\hat{\mathbf{p}}_{AA})\otimes\mathbf{1}_\omega^\mathsf{T}\mathbf{F}'_{aa} \\ \hline
\mathbf{0} & \mathbf{0} & \mathbf{0} & \frac{\alpha}{2\mathrm{p_n}}(\mathbf{F}_{AA}\hat{\mathbf{p}}_{AA})\otimes\mathbf{1}_\omega^\mathsf{T}\mathbf{F}'_{Aa} & \mathbf{0} & \frac{\alpha}{\mathrm{p_n}}(\mathbf{F}_{AA}\hat{\mathbf{p}}_{AA})\otimes\mathbf{1}_\omega^\mathsf{T}\mathbf{F}'_{aa} \\
\mathbf{0} & \mathbf{0} & \mathbf{0} & \frac{(1-\alpha)}{2\mathrm{p_n}}(\mathbf{F}_{AA}\hat{\mathbf{p}}_{AA})\otimes\mathbf{1}_\omega^\mathsf{T}\mathbf{F}'_{Aa} & \mathbf{0} & \frac{(1-\alpha)}{\mathrm{p_n}}(\mathbf{F}_{AA}\hat{\mathbf{p}}_{AA})\otimes\mathbf{1}_\omega^\mathsf{T}\mathbf{F}'_{aa} \\ \hline
\mathbf{0} & \mathbf{0} & \mathbf{0} & \mathbf{0} & \mathbf{0} & \mathbf{0} \\
\mathbf{0} & \mathbf{0} & \mathbf{0} & \mathbf{0} & \mathbf{0} & \mathbf{0}
\end{array}\right) \qquad \text{(A-63)}$$

A.2.3 The Jacobian

Putting it all together, i.e. substituting equations (A-29), (A-34), and (A-63) into equation (A-27), we get the Jacobian (on the next page)

$$
\begin{aligned}
\mathbf{M} &= \frac{1}{\lambda_{AA}} \left(\begin{array}{cc|cc|cc}
\mathbf{U}_{AA} + \alpha \mathbf{F}_{AA} & \mathbf{0} & \frac{1}{2}\alpha \mathbf{F}_{Aa} & \mathbf{0} & \mathbf{0} & \mathbf{0} \\
(1-\alpha)\mathbf{F}_{AA} & \mathbf{U}'_{AA} & \frac{1}{2}(1-\alpha)\mathbf{F}_{Aa} & \mathbf{0} & \mathbf{0} & \mathbf{0} \\
\hline
\mathbf{0} & \mathbf{0} & \mathbf{U}_{Aa} + \frac{1}{2}\alpha \mathbf{F}_{Aa} & \mathbf{0} & \alpha \mathbf{F}_{aa} & \mathbf{0} \\
\mathbf{0} & \mathbf{0} & \frac{1}{2}(1-\alpha)\mathbf{F}_{Aa} & \mathbf{U}'_{Aa} & (1-\alpha)\mathbf{F}_{aa} & \mathbf{0} \\
\hline
\mathbf{0} & \mathbf{0} & \mathbf{0} & \mathbf{0} & \mathbf{U}_{aa} & \mathbf{0} \\
\mathbf{0} & \mathbf{0} & \mathbf{0} & \mathbf{0} & \mathbf{0} & \mathbf{U}'_{aa}
\end{array} \right) \\
&- \frac{1}{\lambda_{AA}} \left(\begin{array}{cc|cc|cc}
\hat{\mathbf{p}}_{AA} \otimes \mathbf{1}_\omega^\intercal \mathbf{U}_{AA} & \hat{\mathbf{p}}_{AA} \otimes \mathbf{1}_\omega^\intercal \mathbf{U}'_{AA} & \hat{\mathbf{p}}_{AA} \otimes \mathbf{1}_\omega^\intercal \mathbf{U}_{Aa} & \hat{\mathbf{p}}_{AA} \otimes \mathbf{1}_\omega^\intercal \mathbf{U}'_{Aa} & \hat{\mathbf{p}}_{AA} \otimes \mathbf{1}_\omega^\intercal \mathbf{U}_{aa} & \hat{\mathbf{p}}_{AA} \otimes \mathbf{1}_\omega^\intercal \mathbf{U}'_{aa} \\
\hat{\mathbf{p}}'_{AA} \otimes \mathbf{1}_\omega^\intercal \mathbf{U}_{AA} & \hat{\mathbf{p}}'_{AA} \otimes \mathbf{1}_\omega^\intercal \mathbf{U}'_{AA} & \hat{\mathbf{p}}'_{AA} \otimes \mathbf{1}_\omega^\intercal \mathbf{U}_{Aa} & \hat{\mathbf{p}}'_{AA} \otimes \mathbf{1}_\omega^\intercal \mathbf{U}'_{Aa} & \hat{\mathbf{p}}'_{AA} \otimes \mathbf{1}_\omega^\intercal \mathbf{U}_{aa} & \hat{\mathbf{p}}'_{AA} \otimes \mathbf{1}_\omega^\intercal \mathbf{U}'_{aa} \\
\hline
\mathbf{0} & \mathbf{0} & \mathbf{0} & \mathbf{0} & \mathbf{0} & \mathbf{0} \\
\mathbf{0} & \mathbf{0} & \mathbf{0} & \mathbf{0} & \mathbf{0} & \mathbf{0} \\
\hline
\mathbf{0} & \mathbf{0} & \mathbf{0} & \mathbf{0} & \mathbf{0} & \mathbf{0} \\
\mathbf{0} & \mathbf{0} & \mathbf{0} & \mathbf{0} & \mathbf{0} & \mathbf{0}
\end{array} \right) \\
&- \frac{1}{\lambda_{AA}} \left(\begin{array}{cc|cc|cc}
\alpha \hat{\mathbf{p}}_{AA} \otimes \mathbf{1}_\omega^\intercal \mathbf{F}_{AA} & \mathbf{0} & \alpha \hat{\mathbf{p}}_{AA} \otimes \mathbf{1}_\omega^\intercal \mathbf{F}_{Aa} & \mathbf{0} & \alpha \hat{\mathbf{p}}_{AA} \otimes \mathbf{1}_\omega^\intercal \mathbf{F}_{aa} & \mathbf{0} \\
(1-\alpha)\hat{\mathbf{p}}'_{AA} \otimes \mathbf{1}_\omega^\intercal \mathbf{F}_{AA} & \mathbf{0} & (1-\alpha)\hat{\mathbf{p}}'_{AA} \otimes \mathbf{1}_\omega^\intercal \mathbf{F}_{Aa} & \mathbf{0} & (1-\alpha)\hat{\mathbf{p}}'_{AA} \otimes \mathbf{1}_\omega^\intercal \mathbf{F}_{aa} & \mathbf{0} \\
\hline
\mathbf{0} & \mathbf{0} & \mathbf{0} & \mathbf{0} & \mathbf{0} & \mathbf{0} \\
\mathbf{0} & \mathbf{0} & \mathbf{0} & \mathbf{0} & \mathbf{0} & \mathbf{0} \\
\hline
\mathbf{0} & \mathbf{0} & \mathbf{0} & \mathbf{0} & \mathbf{0} & \mathbf{0} \\
\mathbf{0} & \mathbf{0} & \mathbf{0} & \mathbf{0} & \mathbf{0} & \mathbf{0}
\end{array} \right) \\
&+ \frac{1}{\lambda_{AA}} \left(\begin{array}{cc|cc|cc}
\mathbf{0} & \mathbf{0} & \mathbf{0} & -\frac{\alpha}{2\mathrm{p_n}}(\mathbf{F}_{AA}\hat{\mathbf{p}}_{AA}) \otimes \mathbf{1}_\omega^\intercal \mathbf{F}'_{Aa} & \mathbf{0} & -\frac{\alpha}{\mathrm{p_n}}(\mathbf{F}_{AA}\hat{\mathbf{p}}_{AA}) \otimes \mathbf{1}_\omega^\intercal \mathbf{F}'_{aa} \\
\mathbf{0} & \mathbf{0} & \mathbf{0} & -\frac{(1-\alpha)}{2\mathrm{p_n}}(\mathbf{F}_{AA}\hat{\mathbf{p}}_{AA}) \otimes \mathbf{1}_\omega^\intercal \mathbf{F}'_{Aa} & \mathbf{0} & -\frac{(1-\alpha)}{\mathrm{p_n}}(\mathbf{F}_{AA}\hat{\mathbf{p}}_{AA}) \otimes \mathbf{1}_\omega^\intercal \mathbf{F}'_{aa} \\
\hline
\mathbf{0} & \mathbf{0} & \mathbf{0} & \frac{\alpha}{2\mathrm{p_n}}(\mathbf{F}_{AA}\hat{\mathbf{p}}_{AA}) \otimes \mathbf{1}_\omega^\intercal \mathbf{F}'_{Aa} & \mathbf{0} & \frac{\alpha}{\mathrm{p_n}}(\mathbf{F}_{AA}\hat{\mathbf{p}}_{AA}) \otimes \mathbf{1}_\omega^\intercal \mathbf{F}'_{aa} \\
\mathbf{0} & \mathbf{0} & \mathbf{0} & \frac{(1-\alpha)}{2\mathrm{p_n}}(\mathbf{F}_{AA}\hat{\mathbf{p}}_{AA}) \otimes \mathbf{1}_\omega^\intercal \mathbf{F}'_{Aa} & \mathbf{0} & \frac{(1-\alpha)}{\mathrm{p_n}}(\mathbf{F}_{AA}\hat{\mathbf{p}}_{AA}) \otimes \mathbf{1}_\omega^\intercal \mathbf{F}'_{aa} \\
\hline
\mathbf{0} & \mathbf{0} & \mathbf{0} & \mathbf{0} & \mathbf{0} & \mathbf{0} \\
\mathbf{0} & \mathbf{0} & \mathbf{0} & \mathbf{0} & \mathbf{0} & \mathbf{0}
\end{array} \right) . \qquad \text{(A-64)}
\end{aligned}
$$

Eigenvalues of the Jacobian

The Jacobian matrix, given by equation (A-64), is upper block triangular, so the eigenvalues of $\mathbf{M}$ are the eigenvalues of the diagonal blocks. The largest absolute eigenvalue of the Jacobian, i.e. the spectral radius $\rho(\mathbf{M})$, determines the stability of the boundary equilibrium. We will denote the three nonzero blocks along the diagonal with $\mathbf{M}_{11}$, $\mathbf{M}_{22}$, and $\mathbf{M}_{33}$ (see equation (17)), such that for example

$$\mathbf{M}_{33} = \frac{1}{\lambda_{AA}} \begin{pmatrix} \mathbf{U}_{aa} & \mathbf{0} \\ \mathbf{0} & \mathbf{U}'_{aa} \end{pmatrix}. \tag{A-65}$$

Block $\mathbf{M}_{33}$ projects perturbations in the aa direction. In the neighbourhood of the AA equilibrium, aa homozygotes are negligibly rare, and thus $\mathbf{M}_{33}$ normally does not determine the stability of $\mathbf{M}$. An exception occurs when

$$\lambda_{AA} < \rho \begin{pmatrix} \mathbf{U}_{aa} & \mathbf{0} \\ \mathbf{0} & \mathbf{U}'_{aa} \end{pmatrix} < 1. \tag{A-66}$$

That is, if the AA population is declining sufficiently rapidly, the aa homozygote may increase in frequency simply by declining to extinction more slowly. If the homozygous AA population is stable or increasing, so that $\lambda_{AA} \geq 1$, this cannot happen. Similarly, if $\mathbf{U}_{aa}$ is age-classified with a maximum age, $\rho(\mathbf{U}_{aa}) = 0$, and the phenomenon can not happen. We neglect this pathological case in our discussions. The block $\mathbf{M}_{11}$ projects perturbations in the AA boundary, and because $\hat{\mathbf{p}}$ is stable to perturbations in that boundary, $\rho(\mathbf{M}_{11}) < 1$.

The stability of $\hat{\mathbf{p}}$ is thus determined by

$$\mathbf{M}_{22} \; = \; \frac{1}{\lambda_{AA}} \begin{pmatrix} \mathbf{U}_{Aa} + \frac{1}{2}\alpha \mathbf{F}_{Aa} & \frac{\alpha}{2} \frac{(\mathbf{F}_{AA}\hat{\mathbf{p}}_{AA}) \otimes (\mathbf{1}_\omega^{\mathsf{T}} \mathbf{F}'_{Aa})}{\mathbf{1}_\omega^{\mathsf{T}} \mathbf{F}'_{AA} \hat{\mathbf{p}}'_{AA}} \\ \frac{1}{2}(1-\alpha)\mathbf{F}_{Aa} & \mathbf{U}'_{Aa} + \frac{(1-\alpha)}{2} \frac{(\mathbf{F}_{AA}\hat{\mathbf{p}}_{AA}) \otimes (\mathbf{1}_\omega^{\mathsf{T}} \mathbf{F}'_{Aa})}{\mathbf{1}_\omega^{\mathsf{T}} \mathbf{F}'_{AA} \hat{\mathbf{p}}'_{AA}} \end{pmatrix}, \tag{A-67}$$

which is equation (18) in the main text. The largest absolute value of the eigenvalues of the Jacobian matrix, the dominant eigenvalue, evaluated at the AA boundary, denoted by $\tilde{\zeta}_{AA}$, is therefore

$$\tilde{\zeta}_{AA} = \frac{1}{\lambda_{AA}} \rho \begin{pmatrix} \mathbf{U}_{Aa} + \frac{1}{2}\alpha \mathbf{F}_{Aa} & \frac{\alpha}{2} \frac{(\mathbf{F}_{AA}\hat{\mathbf{p}}_{AA}) \otimes (\mathbf{1}_\omega^{\mathsf{T}} \mathbf{F}'_{Aa})}{\mathbf{1}_\omega^{\mathsf{T}} \mathbf{F}'_{AA} \hat{\mathbf{p}}'_{AA}} \\ \frac{1}{2}(1-\alpha)\mathbf{F}_{Aa} & \mathbf{U}'_{Aa} + \frac{(1-\alpha)}{2} \frac{(\mathbf{F}_{AA}\hat{\mathbf{p}}_{AA}) \otimes (\mathbf{1}_\omega^{\mathsf{T}} \mathbf{F}'_{Aa})}{\mathbf{1}_\omega^{\mathsf{T}} \mathbf{F}'_{AA} \hat{\mathbf{p}}'_{AA}} \end{pmatrix}, \tag{A-68}$$

equation (21) in the main text. By symmetry, the dominant eigenvalue of the Jacobian matrix evaluated at the aa boundary, denoted by $\tilde{\zeta}_{aa}$, is

$$\tilde{\zeta}_{aa} = \frac{1}{\lambda_{aa}} \rho \begin{pmatrix} \mathbf{U}_{Aa} + \frac{1}{2}\alpha \mathbf{F}_{Aa} & \frac{\alpha}{2} \frac{(\mathbf{F}_{aa}\hat{\mathbf{p}}_{aa}) \otimes (\mathbf{1}_\omega^{\mathsf{T}} \mathbf{F}'_{Aa})}{\mathbf{1}_\omega^{\mathsf{T}} \mathbf{F}'_{aa} \hat{\mathbf{p}}'_{aa}} \\ \frac{1}{2}(1-\alpha)\mathbf{F}_{Aa} & \mathbf{U}'_{Aa} + \frac{(1-\alpha)}{2} \frac{(\mathbf{F}_{aa}\hat{\mathbf{p}}_{aa}) \otimes (\mathbf{1}_\omega^{\mathsf{T}} \mathbf{F}'_{Aa})}{\mathbf{1}_\omega^{\mathsf{T}} \mathbf{F}'_{aa} \hat{\mathbf{p}}'_{aa}} \end{pmatrix}, \tag{A-69}$$

which is equation (22). A boundary equilibrium is unstable to invasion by the other allele if the dominant eigenvalue of the Jacobian evaluated at that equlibrium is larger than one. If both boundaries are unstable, i.e., $\tilde{\zeta}_{AA} > 1$ and $\tilde{\zeta}_{aa} > 1$, then both alleles will coexist. The coexistence conditions are therefore given by

$$\rho \begin{pmatrix} \mathbf{U}_{Aa} + \frac{1}{2}\alpha \mathbf{F}_{Aa} & \frac{\alpha}{2} \frac{(\mathbf{F}_{AA}\hat{\mathbf{p}}_{AA}) \otimes (\mathbf{1}_\omega^{\mathsf{T}} \mathbf{F}'_{Aa})}{\mathbf{1}_\omega^{\mathsf{T}} \mathbf{F}'_{AA} \hat{\mathbf{p}}'_{AA}} \\ \frac{1}{2}(1-\alpha)\mathbf{F}_{Aa} & \mathbf{U}'_{Aa} + \frac{(1-\alpha)}{2} \frac{(\mathbf{F}_{AA}\hat{\mathbf{p}}_{AA}) \otimes (\mathbf{1}_\omega^{\mathsf{T}} \mathbf{F}'_{Aa})}{\mathbf{1}_\omega^{\mathsf{T}} \mathbf{F}'_{AA} \hat{\mathbf{p}}'_{AA}} \end{pmatrix} \; > \; \lambda_{AA}, \tag{A-70}$$

$$\rho \begin{pmatrix} \mathbf{U}_{Aa} + \frac{1}{2}\alpha \mathbf{F}_{Aa} & \frac{\alpha}{2} \frac{(\mathbf{F}_{aa}\hat{\mathbf{p}}_{aa}) \otimes (\mathbf{1}_\omega^{\mathsf{T}} \mathbf{F}'_{Aa})}{\mathbf{1}_\omega^{\mathsf{T}} \mathbf{F}'_{aa} \hat{\mathbf{p}}'_{aa}} \\ \frac{1}{2}(1-\alpha)\mathbf{F}_{Aa} & \mathbf{U}'_{Aa} + \frac{(1-\alpha)}{2} \frac{(\mathbf{F}_{aa}\hat{\mathbf{p}}_{aa}) \otimes (\mathbf{1}_\omega^{\mathsf{T}} \mathbf{F}'_{Aa})}{\mathbf{1}_\omega^{\mathsf{T}} \mathbf{F}'_{aa} \hat{\mathbf{p}}'_{aa}} \end{pmatrix} \; > \; \lambda_{aa}, \tag{A-71}$$

(equations (23) and (24)).

B Coexistence conditions under simplifying assumptions

B.1 $\mathbf{U}_i = \mathbf{U}'_i$

In this section, we consider a simplification that removes sexual dimorphism in survival and transition rates, $\mathbf{U}_i = \mathbf{U}'_i$. We consider block $\mathbf{M}_{22}$ of the Jacobian matrix, equation (A-67)

$$\mathbf{M}_{22} \;=\; \frac{1}{\lambda_{AA}}\begin{pmatrix} \mathbf{U}_{Aa} + \frac{1}{2}\alpha\mathbf{F}_{Aa} & \frac{1}{2}\alpha\mathbf{D}_{AA} \\ \frac{1}{2}(1-\alpha)\mathbf{F}_{Aa} & \mathbf{U}_{Aa} + \frac{1}{2}(1-\alpha)\mathbf{D}_{AA} \end{pmatrix}, \tag{B-1}$$

where we use the following notation (equation (19) the main text),

$$\mathbf{D}_{AA} = \frac{(\mathbf{F}_{AA}\hat{\mathbf{p}}_{AA}) \otimes (\mathbf{1}_\omega^\intercal \mathbf{F}'_{Aa})}{\mathbf{1}_\omega^\intercal \mathbf{F}'_{AA}\hat{\mathbf{p}}'_{AA}}. \tag{B-2}$$

Consider an eigenvector of this matrix,

$$\mathbf{v} = \begin{pmatrix} \mathbf{u} \\ \mathbf{u}' \end{pmatrix}, \tag{B-3}$$

with eigenvalue x, which has to satisfy the following equation

$$\frac{1}{\lambda_{AA}}\begin{pmatrix} \mathbf{U}_{Aa} + \frac{1}{2}\alpha\mathbf{F}_{Aa} & \frac{1}{2}\alpha\mathbf{D}_{AA} \\ \frac{1}{2}(1-\alpha)\mathbf{F}_{Aa} & \mathbf{U}_{Aa} + \frac{1}{2}(1-\alpha)\mathbf{D}_{AA} \end{pmatrix}\begin{pmatrix} \mathbf{u} \\ \mathbf{u}' \end{pmatrix} = x\begin{pmatrix} \mathbf{u} \\ \mathbf{u}' \end{pmatrix}. \tag{B-4}$$

We have written the eigenvector in terms of a vector associated with the female direction, $\mathbf{u}$, and a vector associated with the male direction $\mathbf{u}'$. Write equation (B-4) as two separate equations for the male and female directions,

$$\left(\mathbf{U}_{Aa} + \frac{1}{2}\alpha\mathbf{F}_{Aa}\right)\mathbf{u} + \frac{1}{2}\alpha\mathbf{D}_{AA}\mathbf{u}' \;=\; \lambda_{AA}x\mathbf{u}, \tag{B-5}$$

$$\left(\mathbf{U}_{Aa} + \frac{1}{2}(1-\alpha)\mathbf{D}_{AA}\right)\mathbf{u}' + \frac{1}{2}(1-\alpha)\mathbf{F}_{Aa}\mathbf{u} \;=\; \lambda_{AA}x\mathbf{u}'. \tag{B-6}$$

Moving the terms $\mathbf{U}_{Aa}\mathbf{u}$ and $\mathbf{U}_{Aa}\mathbf{u}'$ to the right in the top and bottom equations respectively yields,

$$\frac{1}{2}\alpha\left[\mathbf{F}_{Aa}\mathbf{u} + \mathbf{D}_{AA}\mathbf{u}'\right] \;=\; \left(\lambda_{AA}x\mathbf{I}_\omega - \mathbf{U}_{Aa}\right)\mathbf{u}, \tag{B-7}$$

$$\frac{1}{2}(1-\alpha)\left[\mathbf{F}_{Aa}\mathbf{u} + \mathbf{D}_{AA}\mathbf{u}'\right] \;=\; \left(\lambda_{AA}x\mathbf{I}_\omega - \mathbf{U}_{Aa}\right)\mathbf{u}'. \tag{B-8}$$

Provided the matrix $(\lambda_{AA}x\mathbf{I} - \mathbf{U}_{Aa})$ is non-singular,

$$\mathbf{u} \;=\; \frac{1}{2}\alpha\left(\lambda_{AA}x\mathbf{I}_\omega - \mathbf{U}_{Aa}\right)^{-1}\left[\mathbf{F}_{Aa}\mathbf{u} + \mathbf{D}_{AA}\mathbf{u}'\right], \tag{B-9}$$

$$\mathbf{u}' \;=\; \frac{1}{2}(1-\alpha)\left(\lambda_{AA}x\mathbf{I}_\omega - \mathbf{U}_{Aa}\right)^{-1}\left[\mathbf{F}_{Aa}\mathbf{u} + \mathbf{D}_{AA}\mathbf{u}'\right], \tag{B-10}$$

which implies

$$\mathbf{u}' = \frac{(1-\alpha)}{\alpha}\mathbf{u}. \tag{B-11}$$

Substituting this relationship between the male and female directions of the eigenvector back into equation (B-4) yields

$$\frac{1}{\lambda_{AA}}\begin{pmatrix} \mathbf{U}_{Aa} + \frac{1}{2}\alpha\mathbf{F}_{Aa} & \frac{\alpha}{2}\mathbf{D}_{AA} \\ \frac{1}{2}(1-\alpha)\mathbf{F}_{Aa} & \mathbf{U}_{Aa} + \frac{(1-\alpha)}{2}\mathbf{D}_{AA} \end{pmatrix}\begin{pmatrix} \mathbf{u} \\ \frac{(1-\alpha)}{\alpha}\mathbf{u} \end{pmatrix} = x\begin{pmatrix} \mathbf{u} \\ \frac{(1-\alpha)}{\alpha}\mathbf{u} \end{pmatrix}. \tag{B-12}$$

Write out the equation for the first block of the eigenvector:

$$\frac{1}{\lambda_{AA}}\left[\mathbf{U}_{Aa}+\frac{1}{2}\alpha\mathbf{F}_{Aa}+\frac{1}{2}(1-\alpha)\mathbf{D}_{AA}\right]\mathbf{u}=x\mathbf{u}. \tag{B-13}$$

This equation shows that the eigenvalues x of the $2\omega \times 2\omega$ matrix $\mathbf{M}_{22}$, given by equation (B-1), are also eigenvalues of the following matrix of dimensions $\omega\times\omega$ (namely, the left-hand side of equation (B-13)),

$$\frac{1}{\lambda_{AA}}\left[\mathbf{U}_{Aa}+\frac{1}{2}\alpha\mathbf{F}_{Aa}+\frac{1}{2}(1-\alpha)\mathbf{D}_{AA}\right]. \tag{B-14}$$

Therefore the dominant eigenvalue of the $2\omega \times 2\omega$ matrix $\mathbf{M}_{22}$ is

$$\tilde{\zeta}_{AA}=\frac{1}{\lambda_{AA}}\rho\left(\mathbf{U}_{Aa}+\frac{1}{2}\alpha\mathbf{F}_{Aa}+\frac{1}{2}(1-\alpha)\mathbf{D}_{AA}\right). \tag{B-15}$$

Similarly for $\tilde{\zeta}_{aa}$,

$$\tilde{\zeta}_{aa}=\frac{1}{\lambda_{aa}}\rho\left(\mathbf{U}_{Aa}+\frac{1}{2}\alpha\mathbf{F}_{Aa}+\frac{1}{2}(1-\alpha)\mathbf{D}_{aa}\right). \tag{B-16}$$

The coexistence conditions are then given by

$$\rho\left(\mathbf{U}_{Aa}+\frac{1}{2}\alpha\mathbf{F}_{Aa}+\frac{1}{2}(1-\alpha)\mathbf{D}_{AA}\right) > \lambda_{AA}, \tag{B-17}$$

$$\rho\left(\mathbf{U}_{Aa}+\frac{1}{2}\alpha\mathbf{F}_{Aa}+\frac{1}{2}(1-\alpha)\mathbf{D}_{aa}\right) > \lambda_{aa}, \tag{B-18}$$

B.2 $\mathbf{U}_i = \mathbf{U}'_i$, $\mathbf{F}_i = \beta\mathbf{F}'_i$ and $\alpha = 0.5$

Next we make the additional simplifying assumption that male mating success is proportional (or equal) to female fertility, $\mathbf{F}'_i = \beta\mathbf{F}_i$. Finally, we also set the primary sex to one, i.e. $\alpha = 0.5$. We will show that these simplifying assumptions reduce the coexistence conditions, equations (A-71) and (A-70) to heterozygote superiority in genotype-specific population growth rate, i.e. $\lambda_{Aa} > \lambda_{AA}$ and $\lambda_{Aa} > \lambda_{aa}$.

Males produce gametes rather than offspring and we assume there is only one type of male gamete. The $\mathbf{F}'_i$ matrices therefore only have one nonzero row. Without loss of generality, we put the newborns in the first stage and hence the first row of the matrix $\mathbf{F}'_i$ is the only nonzero row. Assuming male mating success is proportional to female fertility rates, $\mathbf{F}'_i = \beta\mathbf{F}_i$, then implies that females also only produce one type of offspring. Define a vector of fertilities of dimensions $\omega \times 1$ for each genotype, $\mathbf{f}_i$, such that

$$\begin{aligned}\mathbf{F}_i &= \begin{pmatrix} f_i(1) & \dots & f_i(\omega) \\ 0 & \dots & 0 \\ \vdots & & \vdots \\ \mathbf{0} & \dots & 0 \end{pmatrix}, & \text{(B-19)}\\ &= \mathbf{e}_1 \otimes \mathbf{f}_i^{\mathsf{T}}. & \text{(B-20)}\end{aligned}$$

The following equalities hold in this case

$$\begin{aligned}\mathbf{F}_{AA}\hat{\mathbf{p}}_{AA} &= \mathbf{e}_1 \otimes (\mathbf{f}_{AA}^{\mathsf{T}}\hat{\mathbf{p}}_{AA}), & \text{(B-21)}\\ \mathbf{1}_\omega^{\mathsf{T}}\mathbf{F}'_{AA}\hat{\mathbf{p}}_{AA} &= \beta\mathbf{f}_{AA}^{\mathsf{T}}\hat{\mathbf{p}}_{AA}, & \text{(B-22)}\\ \mathbf{1}_\omega^{\mathsf{T}}\mathbf{F}'_{Aa} &= \beta\mathbf{f}_{Aa}^{\mathsf{T}}. & \text{(B-23)}\end{aligned}$$

Note that $\mathbf{f}_{AA}^{\intercal}\hat{\mathbf{p}}_{AA}$ is a scalar, and that

$$\begin{aligned}
\mathbf{F}_{AA}\hat{\mathbf{p}}_{AA} &= \mathbf{e}_1 \otimes (\mathbf{f}_{AA}^{\intercal}\hat{\mathbf{p}}_{AA}) && \text{(B-24)} \\
&= \begin{pmatrix} \mathbf{f}_{AA}^{\intercal}\hat{\mathbf{p}}_{AA} \\ \mathbf{0} \\ \vdots \\ \mathbf{0} \end{pmatrix}, && \text{(B-25)}
\end{aligned}$$

and therefore

$$\frac{(\mathbf{F}_{AA}\hat{\mathbf{p}}_{AA})}{\mathbf{1}_{\omega}^{\intercal}\mathbf{F}'_{AA}\hat{\mathbf{p}}_{AA}} = \frac{1}{\beta}\mathbf{e}_1 \tag{B-26}$$

Substituting equations (B-21)-(B-23) into the $\mathbf{D}_{AA}$ matrix yields

$$\begin{aligned}
\mathbf{D}_{AA} &= \frac{(\mathbf{F}_{AA}\hat{\mathbf{p}}_{AA}) \otimes \mathbf{1}_{\omega}^{\intercal}\mathbf{F}'_{Aa}}{\mathbf{1}_{\omega}^{\intercal}\mathbf{F}'_{AA}\hat{\mathbf{p}}_{AA}} && \text{(B-27)} \\
&= \frac{1}{\beta}\mathbf{e}_1 \otimes \beta\mathbf{1}_{\omega}^{\intercal}\mathbf{F}'_{Aa} && \text{(B-28)} \\
&= \mathbf{e}_1 \otimes \mathbf{f}_{Aa}^{\intercal}, && \text{(B-29)} \\
&= \mathbf{F}_{Aa}. && \text{(B-30)}
\end{aligned}$$

Similarly,

$$\mathbf{D}_{aa} = \frac{(\mathbf{F}_{aa}\hat{\mathbf{p}}_{aa}) \otimes (\mathbf{1}_{\omega}^{\intercal}\mathbf{F}'_{Aa})}{\mathbf{1}_{\omega}^{\intercal}\mathbf{F}'_{aa}\hat{\mathbf{p}}'_{aa}} = \mathbf{F}_{Aa}, \tag{B-31}$$

Substituting this expression for $\mathbf{D}_{AA}$ back into the coexistence conditions derived in the previous section, equations (B-17) and (B-18) yields

$$\begin{aligned}
\rho\left(\mathbf{U}_{Aa} + \frac{1}{2}\alpha\mathbf{F}_{Aa} + \frac{1}{2}(1-\alpha)\mathbf{F}_{Aa}\right) &> \rho\left(\mathbf{U}_{AA} + \alpha\mathbf{F}_{AA}\right), && \text{(B-32)} \\
\rho\left(\mathbf{U}_{Aa} + \frac{1}{2}\alpha\mathbf{F}_{Aa} + \frac{1}{2}(1-\alpha)\mathbf{F}_{Aa}\right) &> \rho\left(\mathbf{U}_{aa} + \alpha\mathbf{F}_{aa}\right), && \text{(B-33)}
\end{aligned}$$

or

$$\begin{aligned}
\rho\left(\mathbf{U}_{Aa} + \frac{1}{2}\mathbf{F}_{Aa}\right) &> \rho\left(\mathbf{U}_{AA} + \alpha\mathbf{F}_{AA}\right), && \text{(B-34)} \\
\rho\left(\mathbf{U}_{Aa} + \frac{1}{2}\mathbf{F}_{Aa}\right) &> \rho\left(\mathbf{U}_{aa} + \alpha\mathbf{F}_{aa}\right), && \text{(B-35)}
\end{aligned}$$

(equations (29) and (30) in the main text).

If we define heterozygote population growth rate analogously to the two homozygote population growth rates, then

$$\lambda_{Aa} = \rho\left(\mathbf{U}_{Aa} + \alpha\mathbf{F}_{Aa}\right). \tag{B-36}$$

Therefore when $\alpha = \frac{1}{2}$, the coexistence conditions given by equations (B-34) and (B-35) are equal to heterozygote superiority in λ, i.e.

$$\begin{aligned}
\lambda_{Aa} &> \lambda_{AA}, && \text{(B-37)} \\
\lambda_{Aa} &> \lambda_{aa}. && \text{(B-38)}
\end{aligned}$$

B.3 All breeding males have equal mating success ($\mathbf{F}'_i = \mathbf{e}_1 \otimes \mathbf{c}_i^\mathsf{T}$)

Now we discuss a special case of the one-sex model where we assume that breeding males all have the same mating success, independent of their genotype and stage. This model was introduced in de Vries and Caswell (2019). The frequencies in the gamete pool, q_A and q_a, are simply equal to the frequencies of these alleles in the breeding part of the population, denoted by q_A^b and q_a^b in de Vries and Caswell (2019). The mating population is defined by a set of indicator vectors $\mathbf{c}_i$ for $i = 1, \ldots, g$ that show which stages of genotype i take part in mating. That is, the entries of $\mathbf{c}_i$ are 1 if that stage of genotype i reproduces, and 0 otherwise.

The genotype specific mating success matrices $\mathbf{F}'_i$ can then be written in terms of these indicator vectors as follows

$$\mathbf{F}'_i = \mathbf{e}_1 \otimes \mathbf{c}_i^\mathsf{T}. \tag{B-39}$$

Use

$$\begin{aligned} \mathbf{1}_\omega^\mathsf{T}\mathbf{F}'_i &= \mathbf{1}_\omega^\mathsf{T}\left(\mathbf{e}_1 \otimes \mathbf{c}_i^\mathsf{T}\right) && \text{(B-40)} \\ &= \mathbf{c}_i^\mathsf{T}. && \text{(B-41)} \end{aligned}$$

to write

$$\mathbf{1}_\omega^\mathsf{T}\mathbf{F}'_{AA}\hat{\mathbf{p}}_{AA} = \mathbf{c}_{AA}^\mathsf{T}\hat{\mathbf{p}}_{AA} = p_b, \tag{B-42}$$

where p_b is the fraction of the total population that is in a breeding stage. Substitute this expression into the definition of $\mathbf{D}_{AA}$,

$$\begin{aligned} \mathbf{D}_{AA} &= \frac{(\mathbf{F}_{AA}\hat{\mathbf{p}}_{AA}) \otimes \mathbf{1}_\omega^\mathsf{T}\mathbf{F}'_{Aa}}{\mathbf{1}_\omega^\mathsf{T}\mathbf{F}'_{AA}\hat{\mathbf{p}}_{AA}} && \text{(B-43)} \\ &= \frac{1}{p_b}(\mathbf{F}_{AA}\hat{\mathbf{p}}_{AA}) \otimes \mathbf{1}_\omega^\mathsf{T}\mathbf{F}'_{Aa}. && \text{(B-44)} \end{aligned}$$

Use

$$\begin{aligned} \mathbf{1}_\omega^\mathsf{T}\mathbf{F}'_{Aa} &= \mathbf{1}_\omega^\mathsf{T}\left(\mathbf{e}_1 \otimes \mathbf{c}_{Aa}^\mathsf{T}\right) && \text{(B-45)} \\ &= \mathbf{c}_{Aa}^\mathsf{T}, && \text{(B-46)} \end{aligned}$$

to obtain

$$\zeta_{AA} = \frac{1}{\lambda_{AA}}\rho\left(\mathbf{U}_{Aa} + \frac{1}{2}\alpha\mathbf{F}_{Aa} + \frac{1}{2p_b}(1-\alpha)(\mathbf{F}_{AA}\hat{\mathbf{p}}_{AA}) \otimes \mathbf{c}_{Aa}^\mathsf{T}\right), \tag{B-47}$$

and equivalently,

$$\zeta_{aa} = \frac{1}{\lambda_{aa}}\rho\left(\mathbf{U}_{Aa} + \frac{1}{2}\alpha\mathbf{F}_{Aa} + \frac{1}{2p_b}(1-\alpha)(\mathbf{F}_{aa}\hat{\mathbf{p}}_{aa}) \otimes \mathbf{c}_{Aa}^\mathsf{T}\right). \tag{B-48}$$

These two equations correspond exactly with the results reported in de Vries and Caswell (2019) for $\alpha = 0.5$, where α is absorbed into the definition of the fertility matrix in the one-sex model.

B.4 No population structure

In the absence of population structure, the demographic matrices $\mathbf{U}_i$, $\mathbf{U}'_i$, $\mathbf{F}_i$, and $\mathbf{F}'_i$ all reduce to scalars, which we will label as u_i, u'_i, f_i, and f'_i respectively. Figure A1 shows the coexistence conditions for a population without any age or stage structure. The flow diagram follows exactly the same series of simplifications as Figure 3. First male and female survival are equated (Model 2). Next polymorphism conditions are shown for two different simplifying assumptions about fertility and mating success. Model 3A assumes the gene does not affect male mating success. We arbitrarily set male mating success equal to one for all genotypes. Finally model 3B assumes that genotype-specific male mating success is equal or proportional to genotype-specific female fertility. The male parameters drop out of the coexistence conditions for both model 3A and 3B.

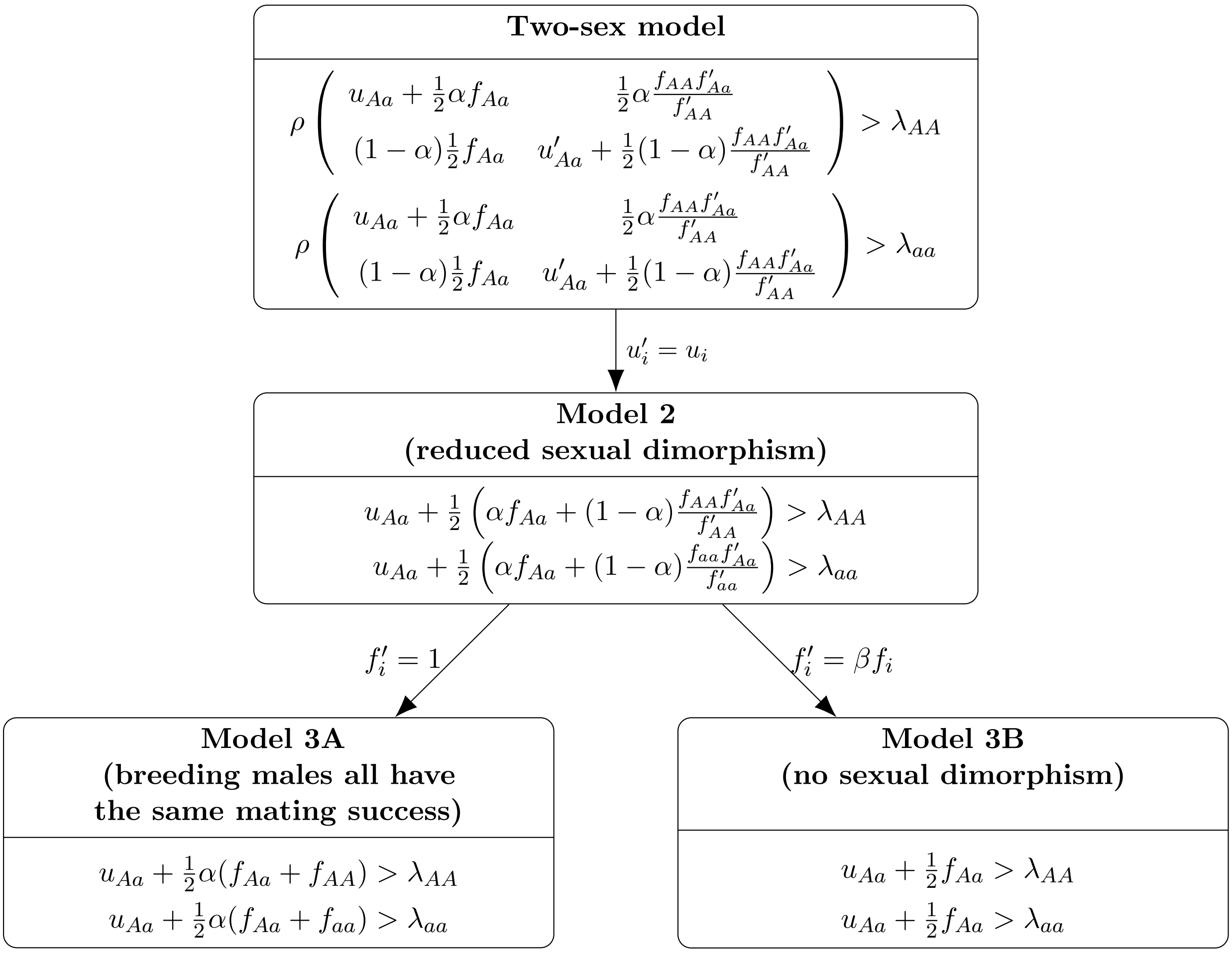

Figure A1: Coexistence conditions for an unstructured two-sex model and several modifications that reduce the extent of sexual dimorphism

C A females-only model (for $\mathbf{U}_i = \mathbf{U}'_i$ and $\alpha = \frac{1}{2}$)

Consider a gene that affects male mating success, or female fertility, or both, in a species with no sexual dimorphism in survival and transition rates. If males and females are furthermore born at equal proportions, then in this Appendix we show that we can model such a population by keeping track of the females only. We show that under above mentioned simplifying assumptions ($\mathbf{U}'_i = \mathbf{U}_i$, $\alpha = \frac{1}{2}$), it is possible to write down a one-sex model that has the same set of equilibria as the two-sex model and the same boundary stability properties as the two-sex model.

Notation: to distinguish between the dominant eigenvalue of the Jacobian in the two-sex model and the one-sex model, we introduce the following notation,

$$\begin{aligned} \tilde{\zeta}_i &= \text{Dominant eigenvalue of the Jacobian evaluated at the } i \text{ boundary of the two-sex model,} \\ \zeta_i &= \text{Dominant eigenvalue of the Jacobian evaluated at the } i \text{ boundary of the one-sex model.} \end{aligned}$$

We start by showing that it is possible to construct a one-sex model with an equilibrium population structure that satisfies the same equation as the equilibrium population structure of the two-sex model when $\mathbf{U}_i = \mathbf{U}'_i$ (assuming such an equilibrium structure exists). Next we verify that the Jacobian of the one-sex model and the Jacobian of the two-sex model have the same dominant eigenvalue at the boundary equilibria, which ensures the models give the same stability conditions for the boundary equilibria.

C.1 Stationary distribution

We start by showing that it is possible to write down a model projecting the female vector only with a stationary distribution that satisfies the same equation as the full two-sex model. First, we write the population projection equation of the two-sex model, equation (3), as two projection equations, one for males and one for females, using equation (7) and using that $\mathcal{U} = \mathcal{U}'$:

$$\begin{aligned} \mathbf{n}(t+1) &= \mathcal{U}\mathbf{n}(t) + \alpha\mathcal{F}\left(\mathbf{p}'\right)\mathbf{n}(t), && \text{(C-1)} \\ \mathbf{n}'(t+1) &= \mathcal{U}\mathbf{n}'(t) + (1-\alpha)\mathcal{F}\left(\mathbf{p}'\right)\mathbf{n}(t). && \text{(C-2)} \end{aligned}$$

A nonlinear version of the Perron-Frobenius theorem guarantees there exists a nontrivial (nonzero) constant population structure which satisfies the following equation,

$$\tilde{\mathbf{A}}\left[\tilde{\hat{\mathbf{n}}}\right]\tilde{\hat{\mathbf{n}}} = \lambda\tilde{\hat{\mathbf{n}}}, \qquad \text{(C-3)}$$

provided the population projection matrix $\tilde{\mathbf{A}}(\tilde{\mathbf{p}})$ is continuous and does not map any nonzero vector directly to zero (Nussbaum, 1986, 1989). The constant population stucture can be written in terms of the sex-specific population vectors as follows,

$$\begin{aligned} \lambda\hat{\mathbf{n}} &= \mathcal{U}\hat{\mathbf{n}} + \alpha\mathcal{F}\left(\hat{\mathbf{p}}'\right)\hat{\mathbf{n}}, && \text{(C-4)} \\ \lambda\hat{\mathbf{n}}' &= \mathcal{U}\hat{\mathbf{n}}' + (1-\alpha)\mathcal{F}\left(\hat{\mathbf{p}}'\right)\hat{\mathbf{n}}. && \text{(C-5)} \end{aligned}$$

A few lines of algebra yield

$$\begin{aligned} \hat{\mathbf{n}} &= \alpha\left(\lambda\mathbf{I} - \mathcal{U}\right)^{-1}\mathcal{F}\left(\hat{\mathbf{p}}'\right)\hat{\mathbf{n}}, && \text{(C-6)} \\ \hat{\mathbf{n}}' &= (1-\alpha)\left(\lambda\mathbf{I} - \mathcal{U}\right)^{-1}\mathcal{F}\left(\hat{\mathbf{p}}'\right)\hat{\mathbf{n}}, && \text{(C-7)} \end{aligned}$$

provided the matrix $(\lambda\mathbf{I} - \mathbf{U})$ is invertible. Equations (C-6) and (C-7) imply that $\hat{\mathbf{n}}' = \frac{(1-\alpha)}{\alpha}\hat{\mathbf{n}}$. Since the male population vector is proportional to the female population vector,

the male frequency vector is equal to the female frequency. We can therefore replace the male frequency vector $\mathbf{p}'$ with the female frequency vector $\mathbf{p}$ in equation (C-4),

$$\lambda\hat{\mathbf{n}} = \left[\mathcal{U} + \alpha\mathcal{F}\left(\hat{\mathbf{p}}\right)\right]\hat{\mathbf{n}}. \tag{C-8}$$

This condition for the female equilibrium population structure is not a function of the male population vector. It is therefore possible to write down a one-sex model with an equilibrium population structure that satisfies the same equation as the equilibrium population structure of the two-sex model, namely:

$$\mathbf{n}(t+1) = \left[\mathcal{U} + \alpha\mathcal{F}\left(\mathbf{p}(t)\right)\right]\mathbf{n}(t), \tag{C-9}$$

where the population vector is

$$\mathbf{n} = \begin{pmatrix} \mathbf{n}_{AA} \\ \mathbf{n}_{Aa} \\ \mathbf{n}_{aa} \end{pmatrix}. \tag{C-10}$$

The fertility matrix is the same as for the two-sex model, except now the frequencies in the gamete pool are calculated from the female population vector, i.e. we replace q'_A and q'_a by q_A and q_a,

$$\begin{pmatrix} q_A \\ q_a \end{pmatrix} = \frac{\mathbf{W}'\mathbf{F}'\mathbf{n}}{\|\mathbf{W}'\mathbf{F}'\mathbf{n}\|}, \tag{C-11}$$

and equation (11) becomes

$$\mathcal{F}(\mathbf{p}) \quad = \quad \begin{pmatrix} q_A\mathbf{F}_{AA} & \frac{1}{2}q_A\mathbf{F}_{Aa} & \mathbf{0} \\ q_a\mathbf{F}_{AA} & \frac{1}{2}\mathbf{F}_{Aa} & q_A\mathbf{F}_{aa} \\ \mathbf{0} & \frac{1}{2}q_a\mathbf{F}_{Aa} & q_a\mathbf{F}_{aa} \end{pmatrix}. \tag{C-12}$$

Although the two models have constant population structures that satisfy the same equation (equation (C-8)), the stability of this equilibrium structure in the two models is not guaranteed to be the same. To check whether the boundary equilibria have the same stability properties in the two models, we check that the dominant eigenvalue of the Jacobian of the one-sex model is indeed equal to the dominant eigenvalue of the two-sex model in section C.2, which turns out to be the case when $\alpha = 0.5$.

C.2 Coexistence conditions in the females-only model (i.e. $\mathbf{U}_i = \mathbf{U}'_i$)

The derivation of the one-sex model is almost identical to the derivation in de Vries and Caswell (2019). The derivations diverge only when dealing with the allele frequencies in the gamete pool and derivatives thereof.

We start from equation (C-9), here repeated

$$\begin{aligned} \mathbf{n}(t+1) &= \left[\mathcal{U} + \alpha\mathcal{F}\left(\mathbf{p}(t)\right)\right]\mathbf{n}(t), && \text{(C-13)} \\ &= \mathbf{A}\left[\mathbf{p}(t)\right]\mathbf{n}(t). && \text{(C-14)} \end{aligned}$$

The population projection matrix can be written in terms of nine $\omega \times \omega$ matrices,

$$\mathbf{A}(\mathbf{p}) = \left(\begin{array}{c|c|c} \mathbf{U}_{AA} + \alpha q_A\mathbf{F}_{AA} & \frac{1}{2}\alpha q_A\mathbf{F}_{Aa} & \mathbf{0} \\ \hline \alpha q_a\mathbf{F}_{AA} & \mathbf{U}_{Aa} + \frac{1}{2}\alpha\mathbf{F}_{Aa} & \alpha q_A\mathbf{F}_{aa} \\ \hline \mathbf{0} & \frac{1}{2}\alpha q_a\mathbf{F}_{Aa} & \mathbf{U}_{aa} + \alpha q_a\mathbf{F}_{aa} \end{array}\right). \tag{C-15}$$

As before, we define the frequency model as follows

$$\mathbf{p}(t+1) = \frac{\mathbf{A}[\mathbf{p}(t)]\mathbf{p}(t)}{\mathbf{1}^{\mathsf{T}}_{g\omega}\mathbf{A}[\mathbf{p}(t)]\mathbf{p}(t)}, \tag{C-16}$$

The Jacobian matrix is obtained by differentiating equation (C-16) and evaluating the resulting derivative at the boundary equilibrium,

$$\mathbf{M} = \left. \frac{\mathrm{d}\mathbf{p}(t+1)}{\mathrm{d}\mathbf{p}^{\mathsf{T}}(t)} \right|_{\hat{\mathbf{p}}} . \tag{C-17}$$

In de Vries and Caswell (2019) it is shown that this method yields the following expression for the Jacobian matrix:

$$\begin{aligned} \mathbf{M} \;=\; & \underbrace{\frac{1}{\lambda_{AA}} \mathbf{A}[\hat{\mathbf{p}}]}_{\textcircled{A}} - \underbrace{\frac{1}{\lambda_{AA}^2} (\hat{\mathbf{p}}^{\mathsf{T}} \otimes \mathbf{I}_{\omega g}) (\mathrm{vec}\mathbf{A}) \left(\mathbf{1}_{\omega g}^{\mathsf{T}} \mathbf{A} \right)}_{\textcircled{B}} \\ + \; & \underbrace{\frac{1}{\lambda_{AA}} (\hat{\mathbf{p}}^{\mathsf{T}} \otimes \mathbf{I}_{\omega g}) \left. \frac{\partial \mathrm{vec}\mathbf{A}}{\partial \mathbf{p}^{\mathsf{T}}} \right|_{\hat{\mathbf{p}}}}_{\textcircled{C}} , \end{aligned} \tag{C-18}$$

where we have identified the three terms as $\textcircled{A}$, $\textcircled{B}$, and $\textcircled{C}$.

The next task is to work out all the terms in the above expression for the Jacobian. For $\textcircled{A}$ and $\textcircled{B}$ we can simply use the results derived in de Vries and Caswell (2019),

$$\textcircled{A} \;=\; \frac{1}{\lambda_{AA}} \left(\begin{array}{c|c|c} \mathbf{U}_{AA} + \alpha \mathbf{F}_{AA} & \frac{1}{2} \alpha \mathbf{F}_{Aa} & \mathbf{0} \\ \hline \mathbf{0} & \mathbf{U}_{Aa} + \frac{1}{2} \alpha \mathbf{F}_{Aa} & \alpha \mathbf{F}_{aa} \\ \hline \mathbf{0} & \mathbf{0} & \mathbf{U}_{aa} \end{array} \right) , \tag{C-19}$$

$$\textcircled{B} = -\frac{1}{\lambda_{AA}} \left(\begin{array}{c|c|c} \hat{\mathbf{p}}_{AA} \otimes \mathbf{1}_{\omega}^{\mathsf{T}} (\mathbf{U}_{AA} + \alpha \mathbf{F}_{AA}) & \hat{\mathbf{p}}_{AA} \otimes \mathbf{1}_{\omega}^{\mathsf{T}} (\mathbf{U}_{Aa} + \alpha \mathbf{F}_{Aa}) & \hat{\mathbf{p}}_{AA} \otimes \mathbf{1}_{\omega}^{\mathsf{T}} (\mathbf{U}_{aa} + \alpha \mathbf{F}_{aa}) \\ \hline \mathbf{0} & \mathbf{0} & \mathbf{0} \\ \hline \mathbf{0} & \mathbf{0} & \mathbf{0} \end{array} \right) . \tag{C-20}$$

For term $\textcircled{C}$ we can use the following result, where we have replaced q_A^b and q_a^b from de Vries and Caswell (2019) with q_A and q_a,

$$\textcircled{C} = \frac{1}{\lambda_{AA}} \left(\begin{array}{c|c|c} \alpha(\mathbf{F}_{AA} \hat{\mathbf{p}}_{AA}) \otimes \frac{\partial q_A}{\partial \mathbf{p}_{AA}^{\mathsf{T}}} & \alpha(\mathbf{F}_{AA} \hat{\mathbf{p}}_{AA}) \otimes \frac{\partial q_A}{\partial \mathbf{p}_{Aa}^{\mathsf{T}}} & \alpha(\mathbf{F}_{AA} \hat{\mathbf{p}}_{AA}) \otimes \frac{\partial q_A}{\partial \mathbf{p}_{aa}^{\mathsf{T}}} \\ \hline -\alpha(\mathbf{F}_{AA} \hat{\mathbf{p}}_{AA}) \otimes \frac{\partial q_A}{\partial \mathbf{p}_{AA}^{\mathsf{T}}} & -\alpha(\mathbf{F}_{AA} \hat{\mathbf{p}}_{AA}) \otimes \frac{\partial q_A}{\partial \mathbf{p}_{Aa}^{\mathsf{T}}} & -\alpha(\mathbf{F}_{AA} \hat{\mathbf{p}}_{AA}) \otimes \frac{\partial q_A}{\partial \mathbf{p}_{aa}^{\mathsf{T}}} \\ \hline \mathbf{0} & \mathbf{0} & \mathbf{0} \end{array} \right) . \tag{C-21}$$

Equation (C-21) requires the derivative of the frequency of allele A in the gamete pool with respect to the population frequency vector:

$$\left. \frac{\partial q_A}{\partial \mathbf{p}^{\mathsf{T}}} \right|_{\hat{\mathbf{p}}} . \tag{C-22}$$

Start with equation (C-11):

$$\begin{pmatrix} q_A \\ q_a \end{pmatrix} = \frac{\mathbf{W}' \mathbf{F}' \mathbf{n}}{\| \mathbf{W}' \mathbf{F}' \mathbf{n} \|} , \tag{C-23}$$

therefore

$$q_A = \frac{\mathbf{e}_1^{\mathsf{T}} \mathbf{W}' \mathbf{F}' \mathbf{p}}{\mathbf{1}_2^{\mathsf{T}} \mathbf{W}' \mathbf{F}' \mathbf{p}} , \tag{C-24}$$

where we can substitute $\mathbf{p}$ for $\mathbf{n}$ because of homogeneity and where the one norm can be replaced by $\mathbf{1}_2^{\mathsf{T}} \mathbf{W}' \mathbf{F}' \mathbf{p}$ because $\mathbf{p}$ is nonnegative. For convenience, we will denote the normalizing factor in the denominator with $\mathrm{p_n}$,

$$\mathrm{p_n} = \mathbf{1}_2^{\mathsf{T}} \mathbf{W}' \mathbf{F}' \mathbf{p} \tag{C-25}$$

Taking the derivative of q_A yields

$$\frac{\partial q_A}{\partial \mathbf{p}^\intercal} \;=\; \frac{1}{\mathrm{p_n}}\mathbf{e}_1^\intercal \mathbf{W}'\mathbf{F}'\frac{\partial \mathbf{p}}{\partial \mathbf{p}^\intercal} - \frac{\mathbf{e}_1^\intercal \mathbf{W}'\mathbf{F}'\mathbf{p}}{\mathrm{p_n^2}}\left(\mathbf{1}_2^\intercal \mathbf{W}'\mathbf{F}'\frac{\partial \mathbf{p}}{\partial \mathbf{p}^\intercal}\right). \tag{C-26}$$

Evaluate this expression at the boundary, where

$$\mathrm{p_n} = \mathbf{1}_\omega^\intercal \mathbf{F}'_{AA}\hat{\mathbf{p}}_{AA}, \tag{C-27}$$

to obtain

$$\left.\frac{\partial q_A}{\partial \mathbf{p}^\intercal}\right|_{\hat{\mathbf{p}}} \;=\; \frac{1}{\mathrm{p_n}}\left(\mathbf{0}, -\frac{1}{2}\mathbf{1}_\omega^\intercal \mathbf{F}'_{Aa}, -\mathbf{1}_\omega^\intercal \mathbf{F}'_{aa}\right). \tag{C-28}$$

Finally substituting equation (C-28) into equation (C-21) yields

$$\textcircled{\scriptsize C} = \frac{1}{\lambda_{AA}}\left(\begin{array}{c|c|c} \mathbf{0} & -\frac{1}{2}\alpha\frac{(\mathbf{F}_{AA}\hat{\mathbf{p}}_{AA})\otimes \mathbf{1}_\omega^\intercal \mathbf{F}'_{Aa}}{\mathbf{1}_\omega^\intercal \mathbf{F}'_{AA}\hat{\mathbf{p}}_{AA}} & -\alpha\frac{(\mathbf{F}_{AA}\hat{\mathbf{p}}_{AA})\otimes \mathbf{1}_\omega^\intercal \mathbf{F}'_{aa}}{\mathbf{1}_\omega^\intercal \mathbf{F}'_{AA}\hat{\mathbf{p}}_{AA}} \\ \hline \mathbf{0} & \frac{1}{2}\alpha\frac{(\mathbf{F}_{AA}\hat{\mathbf{p}}_{AA})\otimes \mathbf{1}_\omega^\intercal \mathbf{F}'_{Aa}}{\mathbf{1}_\omega^\intercal \mathbf{F}'_{AA}\hat{\mathbf{p}}_{AA}} & \alpha\frac{(\mathbf{F}_{AA}\hat{\mathbf{p}}_{AA})\otimes \mathbf{1}_\omega^\intercal \mathbf{F}'_{aa}}{\mathbf{1}_\omega^\intercal \mathbf{F}'_{AA}\hat{\mathbf{p}}_{AA}} \\ \hline \mathbf{0} & \mathbf{0} & \mathbf{0} \end{array}\right). \tag{C-29}$$

Putting it all together, i.e. substituting equations (C-19), (C-20), and (C-29) into equation (A-27), we get the following Jacobian:

$$\begin{aligned} \mathbf{M} \;=\; & \frac{1}{\lambda_{AA}}\left(\begin{array}{c|c|c} \mathbf{U}_{AA}+\alpha\mathbf{F}_{AA} & \frac{1}{2}\alpha\mathbf{F}_{Aa} & \mathbf{0} \\ \hline \mathbf{0} & \mathbf{U}_{Aa}+\frac{1}{2}\alpha\mathbf{F}_{Aa} & \alpha\mathbf{F}_{aa} \\ \hline \mathbf{0} & \mathbf{0} & \mathbf{U}_{aa} \end{array}\right) \\ & -\frac{1}{\lambda_{AA}}\left(\begin{array}{c|c|c} \hat{\mathbf{p}}_{AA}\otimes \mathbf{1}_\omega^\intercal(\mathbf{U}_{AA}+\alpha\mathbf{F}_{AA}) & \hat{\mathbf{p}}_{AA}\otimes \mathbf{1}_\omega^\intercal(\mathbf{U}_{Aa}+\alpha\mathbf{F}_{Aa}) & \hat{\mathbf{p}}_{AA}\otimes \mathbf{1}_\omega^\intercal(\mathbf{U}_{aa}+\alpha\mathbf{F}_{aa}) \\ \hline \mathbf{0} & \mathbf{0} & \mathbf{0} \\ \hline \mathbf{0} & \mathbf{0} & \mathbf{0} \end{array}\right) \\ & +\frac{1}{\lambda_{AA}}\left(\begin{array}{c|c|c} \mathbf{0} & -\frac{1}{2}\alpha\frac{(\mathbf{F}_{AA}\hat{\mathbf{p}}_{AA})\otimes \mathbf{1}_\omega^\intercal \mathbf{F}'_{Aa}}{\mathbf{1}_\omega^\intercal \mathbf{F}'_{AA}\hat{\mathbf{p}}_{AA}} & -\alpha\frac{(\mathbf{F}_{AA}\hat{\mathbf{p}}_{AA})\otimes \mathbf{1}_\omega^\intercal \mathbf{F}'_{aa}}{\mathbf{1}_\omega^\intercal \mathbf{F}'_{AA}\hat{\mathbf{p}}_{AA}} \\ \hline \mathbf{0} & \frac{1}{2}\alpha\frac{(\mathbf{F}_{AA}\hat{\mathbf{p}}_{AA})\otimes \mathbf{1}_\omega^\intercal \mathbf{F}'_{Aa}}{\mathbf{1}_\omega^\intercal \mathbf{F}'_{AA}\hat{\mathbf{p}}_{AA}} & \alpha\frac{(\mathbf{F}_{AA}\hat{\mathbf{p}}_{AA})\otimes \mathbf{1}_\omega^\intercal \mathbf{F}'_{aa}}{\mathbf{1}_\omega^\intercal \mathbf{F}'_{AA}\hat{\mathbf{p}}_{AA}} \\ \hline \mathbf{0} & \mathbf{0} & \mathbf{0} \end{array}\right) \end{aligned} \tag{C-30}$$

Eigenvalues of the Jacobian

The Jacobian matrix, given by equation (C-30), is upper block triangular, so the eigenvalues of $\mathbf{M}$ are the eigenvalues of the diagonal blocks. The largest absolute eigenvalue of the Jacobian, i.e. the spectral radius $\rho(\mathbf{M})$, determines the stability of the boundary equilibrium. We will denote the three nonzero blocks along the diagonal with $\mathbf{M}_{11}$, $\mathbf{M}_{22}$, and $\mathbf{M}_{33}$, such that for example

$$\mathbf{M}_{33} = \frac{1}{\lambda_{AA}}\mathbf{U}_{aa}. \tag{C-31}$$

Block $\mathbf{M}_{33}$ projects perturbations in the aa direction but close to the equilibrium, aa homozygotes are negligible to first order. The block $\mathbf{M}_{11}$ projects perturbations in the AA boundary, and because $\hat{\mathbf{p}}$ is stable to perturbations in that boundary, $\rho(\mathbf{M}_{11}) < 1$.

The stability of $\hat{\mathbf{p}}$ is thus determined by

$$\mathbf{M}_{22} \;=\; \frac{1}{\lambda_{AA}}\left(\mathbf{U}_{Aa} + \frac{1}{2}\alpha\mathbf{F}_{Aa} + \frac{1}{2}\alpha\frac{(\mathbf{F}_{AA}\hat{\mathbf{p}}_{AA})\otimes \mathbf{1}_\omega^\intercal \mathbf{F}'_{Aa}}{\mathbf{1}_\omega^\intercal \mathbf{F}'_{AA}\hat{\mathbf{p}}_{AA}}\right). \tag{C-32}$$

The largest absolute value of the eigenvalues of the Jacobian matrix, the dominant eigenvalue, evaluated at the AA boundary, denoted by ζ_{AA}, is therefore

$$\zeta_{AA} = \frac{1}{\lambda_{AA}} \rho \left(\mathbf{U}_{Aa} + \frac{1}{2}\alpha \mathbf{F}_{Aa} + \frac{1}{2}\alpha \frac{(\mathbf{F}_{AA}\hat{\mathbf{p}}_{AA}) \otimes \mathbf{1}_\omega^\intercal \mathbf{F}'_{Aa}}{\mathbf{1}_\omega^\intercal \mathbf{F}'_{AA}\hat{\mathbf{p}}_{AA}} \right). \tag{C-33}$$

By symmetry, the dominant eigenvalue of the Jacobian matrix evaluated at the aa boundary, denoted by ζ_{aa}, is

$$\zeta_{aa} = \frac{1}{\lambda_{aa}} \rho \left(\mathbf{U}_{Aa} + \frac{1}{2}\alpha \mathbf{F}_{Aa} + \frac{1}{2}\alpha \frac{(\mathbf{F}_{aa}\hat{\mathbf{p}}_{aa}) \otimes \mathbf{1}_\omega^\intercal \mathbf{F}'_{Aa}}{\mathbf{1}_\omega^\intercal \mathbf{F}'_{aa}\hat{\mathbf{p}}_{aa}} \right). \tag{C-34}$$

If both boundaries are unstable, then both alleles will coexist. The conditions for a genetic polymorphism are therefore given by

$$\rho \left(\mathbf{U}_{Aa} + \frac{1}{2}\alpha \mathbf{F}_{Aa} + \frac{1}{2}\alpha \frac{(\mathbf{F}_{AA}\hat{\mathbf{p}}_{AA}) \otimes \mathbf{1}_\omega^\intercal \mathbf{F}'_{Aa}}{\mathbf{1}_\omega^\intercal \mathbf{F}'_{AA}\hat{\mathbf{p}}_{AA}} \right) > \lambda_{AA}, \tag{C-35}$$

$$\rho \left(\mathbf{U}_{Aa} + \frac{1}{2}\alpha \mathbf{F}_{Aa} + \frac{1}{2}\alpha \frac{(\mathbf{F}_{aa}\hat{\mathbf{p}}_{aa}) \otimes \mathbf{1}_\omega^\intercal \mathbf{F}'_{Aa}}{\mathbf{1}_\omega^\intercal \mathbf{F}'_{aa}\hat{\mathbf{p}}_{aa}} \right) > \lambda_{aa}. \tag{C-36}$$

Compare this to the polymorphism conditions from the two-sex model when $\mathbf{U}_i = \mathbf{U}'_i$, equations (B-17) and (B-18), here repeated for convenience

$$\rho \left[\mathbf{U}_{Aa} + \frac{1}{2}\alpha \mathbf{F}_{Aa} + \frac{1}{2}(1-\alpha) \frac{(\mathbf{F}_{AA}\hat{\mathbf{p}}_{AA}) \otimes \mathbf{1}_\omega^\intercal \mathbf{F}'_{Aa}}{\mathbf{1}_\omega^\intercal \mathbf{F}'_{AA}\hat{\mathbf{p}}_{AA}} \right] > \lambda_{AA}, \tag{C-37}$$

$$\rho \left[\mathbf{U}_{Aa} + \frac{1}{2}\alpha \mathbf{F}_{Aa} + \frac{1}{2}(1-\alpha) \frac{(\mathbf{F}_{aa}\hat{\mathbf{p}}_{aa}) \otimes \mathbf{1}_\omega^\intercal \mathbf{F}'_{Aa}}{\mathbf{1}_\omega^\intercal \mathbf{F}'_{aa}\hat{\mathbf{p}}_{aa}} \right] > \lambda_{aa}. \tag{C-38}$$

These two sets of coexistence conditions are identical when $1 - \alpha = \alpha$, i.e. when $\alpha = 0.5$.

References

Caswell, H. (2001). *Matrix Population Models: Construction, Analysis, and Interpretation. Second edition.* Sinauer Associates, Sunderland, Massachusetts, USA.

Caswell, H. (2007). Sensitivity analysis of transient population dynamics. *Ecology letters*, 10(1):1–15.

de Vries, C. and Caswell, H. (2019). Stage-structured evolutionary demography: linking life histories, population genetics, and ecological dynamics. *The American Naturalist*, 193(4):545–559.

Magnus, J. R. and Neudecker, H. (1985). Matrix differential calculus with applications to simple, hadamard, and kronecker products. *Journal of Mathematical Psychology*, 29(4):474–492.

Nussbaum, R. D. (1986). Convexity and log convexity for the spectral radius. *Linear Algebra and its Applications*, 73:59–122.

Nussbaum, R. D. (1989). *Iterated nonlinear maps and Hilbert's projective metric. Part II*, volume 401. American Mathematical Soc.

Roth, W. (1934). On direct product matrices. *Bulletin of the American Mathematical Society*, 40:461–468.
